# Mechanotransduction and Stiffness-Sensing: Mechanisms and Opportunities to Control Multiple Molecular Aspects of Cell Phenotype as a Design Cornerstone of Cell-Instructive Biomaterials for Articular Cartilage Repair

**DOI:** 10.3390/ijms21155399

**Published:** 2020-07-29

**Authors:** Mischa Selig, Jasmin C. Lauer, Melanie L. Hart, Bernd Rolauffs

**Affiliations:** 1 G.E.R.N. Research Center for Tissue Replacement, Regeneration & Neogenesis, Department of Orthopedics and Trauma Surgery, Faculty of Medicine, Medical Center—Albert-Ludwigs-University of Freiburg, 79085 Freiburg im Breisgau, Germany; mischa.selig@uniklinik-freiburg.de (M.S.); jasmin.lauer@uniklinik-freiburg.de (J.C.L.); melaniehar@gmail.com (M.L.H.); 2 Faculty of Biology, University of Freiburg, Schaenzlestrasse 1, D-79104 Freiburg, Germany

**Keywords:** mechanotransduction, stiffness sensing, mesenchymal stromal cells (MSCs), chondrocyte, articular cartilage, osteoarthritis, cell shape, immunomodulation, phenotype modulation, de-differentiation, re-differentiation, biomaterials, cartilage repair, clinical, TGF-β, Rho-GTPases, Wnt, α-catenin, β-catenin, SRY-related HMG box gene 9 (SOX9), RhoA/Rho associated protein kinase (ROCK)

## Abstract

Since material stiffness controls many cell functions, we reviewed the currently available knowledge on stiffness sensing and elucidated what is known in the context of clinical and experimental articular cartilage (AC) repair. Remarkably, no stiffness information on the various biomaterials for clinical AC repair was accessible. Using mRNA expression profiles and morphology as surrogate markers of stiffness-related effects, we deduced that the various clinically available biomaterials control chondrocyte (CH) phenotype well, but not to equal extents, and only in non-degenerative settings. Ample evidence demonstrates that multiple molecular aspects of CH and mesenchymal stromal cell (MSC) phenotype are susceptible to material stiffness, because proliferation, migration, lineage determination, shape, cytoskeletal properties, expression profiles, cell surface receptor composition, integrin subunit expression, and nuclear shape and composition of CHs and/or MSCs are stiffness-regulated. Moreover, material stiffness modulates MSC immuno-modulatory and angiogenic properties, transforming growth factor beta 1 (TGF-β1)-induced lineage determination, and CH re-differentiation/de-differentiation, collagen type II fragment production, and TGF-β1- and interleukin 1 beta (IL-1β)-induced changes in cell stiffness and traction force. We then integrated the available molecular signaling data into a stiffness-regulated CH phenotype model. Overall, we recommend using material stiffness for controlling cell phenotype, as this would be a promising design cornerstone for novel future-oriented, cell-instructive biomaterials for clinical high-quality AC repair tissue.

## 1. Introduction

Microenvironmental stimuli control cell fate and function [1]. One of the key biomechanical determinants is the stiffness of the extracellular matrix (ECM) [2,3], which is the scaffolding structure for tissues and organs that embeds the tissue-resident cells. How biophysical forces like stiffness are sensed by cells is investigated in the field of mechanobiology [4], in which mechanotransduction studies unravel how these external forces and the intracellular forces are together converted into biochemical signals and cellular responses [5].

Articular cartilage (AC) is a specialized tissue [6] which primarily consists of water, collagen type II, proteoglycans, and other non-collagenous proteins and glycoproteins [7,8]. The chondrocytes (CHs) are the resident cells that build and maintain the AC matrix by synthesizing new ECM components. The CHs are present in both healthy [9,10,11] and degenerative AC [9,10,12,13]. Osteoarthritis (OA) is a degenerative disease that affects the whole joint, including the AC, subchondral bone, synovial tissues as well as the menisci. A hallmark of this disease is a change in ECM stiffness [14,15], which has been associated with an altered composition of the AC matrix [16], based on a lower proteoglycan synthesis rate, changes in the content and synthesis of the ECM collagen types [17], an “unbundling” of prototypic collagen fibrils [18], and damage to the collagen network with subsequent proteoglycan depletion [19]. The underlying correlations between ECM composition and the mechanical properties of AC have been explored in detail for healthy, developing, degenerating, and post-injurious AC [20,21,22,23,24,25,26,27,28,29]. Based on OA-related changes in ECM stiffness, a number of studies have subsequently examined how biomechanical stiffness influences CH morphology and phenotype. However, even after a decade of mechanobiological research, it remains poorly understood how OA-associated ECM stiffness changes affect CH phenotype and, thus, alter cell behavior during disease progression. 

Therefore, the aim of this review is to summarize how cells and specifically CHs and mesenchymal stem cells (MSCs) sense stiffness, and to answer whether the approach to control material stiffness for controlling cell fate is effective in controlling the phenotype and differentiation of CHs and MSCs, as these are key cells involved in AC repair [30]. Secondly, we aimed to answer if or how the current designs of clinically used biomaterials for AC repair account for utilizing material stiffness in this context, and whether using material stiffness as a cue for controlling cell phenotype would be a promising design cornerstone for novel future-oriented, cell-instructive biomaterials for clinical high-quality AC repair tissue. Overall, this review presents the available data on specific stiffness-related topics in dedicated chapters, whereas the discussion chapter focuses on interpreting these data and assembling a model of the material stiffness-dependency of CH phenotype.

## 2. Clinical Use of CHs and MSCs in AC Repair Procedures

CHs are used for autologous chondrocyte implantation (ACI), which is an established and well-accepted procedure for the treatment of large, localized full-thickness AC defects in both the knee and ankle joints [31,32,33,34]. Microfracture, which is one of the most commonly performed surgical AC repair procedures, relies on the influx of MSCs from the surgically penetrated subchondral bone, to initiate (fibro-)cartilaginous repair [35] of small localized AC defects [31]. Moreover, MSCs are being used in an emerging clinical procedure termed “autologous matrix-induced chondrogenesis” (AMIC™), which, like microfracture, utilizes the influx of MSCs from the surgically penetrated subchondral bone, but in conjunction with administration of a collagen type I/III membrane [36,37].

## 3. Overview: How Do Cells Sense Their Environment?

The ECM provides structural tissue integrity, tissue boundaries, and initiates mechano-sensitive signaling pathways within the attached cells, which then influence cell spreading, migration, proliferation, cell cycle progression, and stem cell differentiation [2,38,39]. Each tissue has its characteristic stiffness, which is the structural property, to which a scaffold, or substrate resists deformation in response to an applied force [40]. This stiffness is determined by the molecular composition and arrangement of the ECM and its measurement depends on its physical dimensions and boundary conditions, whereas the elastic modulus is a material property. Material stiffness can vary across multiple length scales such as the nano-, micro-, and millimeter scales [41] but each tissue has a characteristic stiffness that serves its physiological needs. Soft tissues like the brain have a relatively low elastic modulus of approximately 1 kPa [42], while muscle tissue have an intermediate elastic modulus of roughly 10 kPa [43], whereas AC exhibits an elastic modulus of 70 kPa [28] and more rigid structures like bone exhibit a relatively high elastic modulus of 100 kPa [44]. Cells sense and respond to changes of the ECM stiffness, which is a process that is termed ‘mechanosensing‘ in mechanobiology. In the following text sections, we review proteins and structures such as focal adhesions (FAs), integrins, FA focal complex, stress fibers, Rho GTPases, and focal adhesion kinases that are involved in stiffness sensing.

## 4. Stiffness Sensing 

Mechanical sensing occurs through probing and contraction of actin fibers, which pull and deform the surrounding ECM. Cells exert contraction forces onto their substrate and subsequently adjust their cell-ECM adhesion strength through changes in FA composition and size, and through their cytoskeletal re-arrangements [2] by modulating endogenous cytoskeleton contractility [45]. This leads to a homeostasis in the forces between intracellular forces due to cytoskeletal contractility and extracellular forces that resist this contractility through the stiffness of the ECM. In this context, multiple studies have focused on the mechanosensory mechanisms that range from the behavior of individual proteins or protein assemblies such as stretch-sensitive ion channels and adhesion complexes to mechanisms such as actin cytoskeleton remodeling on a more cellular scale [46]. Although a complete picture has yet to be generated, it is thought that stiffness sensing is mediated by a kinetic mechanism, in which the loading rate on the integrin-actin linkage, which is the connection between the ECM-cell interface and the cytoskeleton, varies [47]. While cells exert traction forces on the substrate, tension across this linkage is increased. On softer substrates, substrate movement might reduce the loading rate on the integrin-actin linkage and force increases slowly, whereas stiff surfaces with relatively low substrate movement might increase the loading rate on the integrin-actin linkage, which then leads to overall FA strengthening [47].

## 5. Proteins and Structures Involved in Stiffness Sensing

### 5.1. Focal Adhesions

A key structure involved in force sensing and the activation of mechanotransduction pathways is the FA. FAs physically connect the actin cytoskeleton to the ECM [48,49,50]. Moreover, FAs are dynamic structures because changes in the substrate affect FA size and composition through molecular assembly and disassembly by protein turnover [51,52,53,54]. Thus, cells grown on stiffer substrates have larger and more stable FAs, with increased F-actin assembly and increased adhesion [43], whereas cells on softer substrates display small and punctuated but still dynamic FAs [55,56]. 

**Figure 1:**
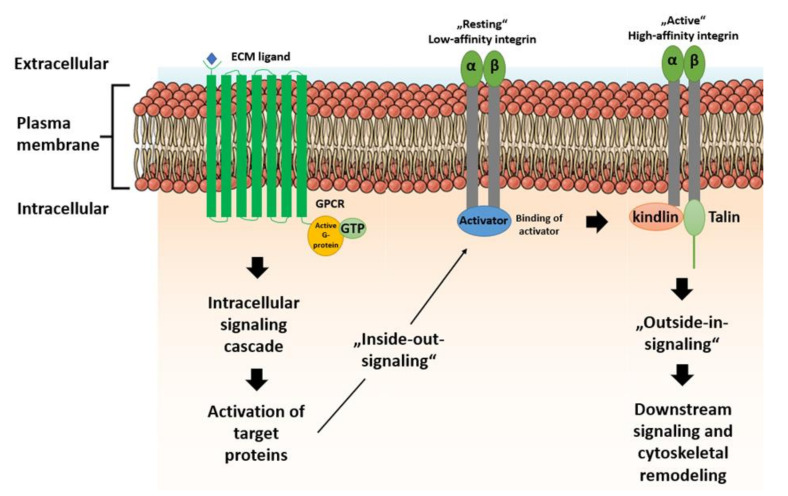
Inside-out and outside-in signaling. Binding of an extracellular matrix (ECM) ligand induces activation of the G-protein receptor. The active G protein initiates an intracellular signaling cascade to activate downstream effectors such as activator proteins, which turn the integrins from a resting to an active state, and which sets up the binding of talin and kindlin to the cytoplasmic integrin domains. This induces outside-in signaling and initiates further downstream processes for subsequent cytoskeletal remodeling.

The FA structure has a defined nano-scale architecture, in which the ‘integrin signaling layer’ (Figure 1) contains the cell’s integrins. In this layer, paxillin and focal adhesion kinase (FAK) localize with the integrin cytoplasmic tails [57,58] in order to recruit further signaling molecules and to regulate actin cytoskeleton remodeling. Paxillin is a multi-domain adaptor between the plasma membrane and the actin cytoskeleton [59] that serves as a docking protein for recruiting signaling molecules [60]. FAK is a cytoplasmic tyrosine kinase that localizes to FAs and regulates actin cytoskeleton remodeling for integrin-mediated processes such as cell spreading and migration [61], for example, by participating in actin polymerization [62,63]. The ‘force transduction layer’ contains mechanosensitive proteins such as talin and vinculin [64,65], which play key roles in mechanotransduction, as recent evidence suggests that talin is the key force-sensing molecule and vinculin one of the key mechanoeffectors [66]. Talin links integrins directly to actin, regulates integrin adhesion strength, increases FA size in response to force, and increases the affinity of integrin for ligands [66]. Moreover, upon mechanical stretching of the molecule talin exposes otherwise not accessible binding sites for the recruitment of additional FA proteins such as vinculin. Vinculin recruitment reinforces the FAs, as recruited vinculin crosslinks an actin filament to the talin molecule [67]. This binding of talin to actin filaments by vinculin or other FA forming molecules upon talin stretching is a critical step in mechanically linking the cell and ECM [68]. Recently, it has been reported that talin in the FAs is under tension, that vinculin increases talin tension, and that softer substrates decrease talin tension [47]. This appears relevant, as local talin tension correlates with F-actin stress fiber alignment [69] and recent evidence suggests a complex relationship between talin tension, actin/vinculin localization, local actin organization, and FA dynamics [69]. Subsequently, talin-depleted cells have a decreased ability to stiffen in response to generated tension [70,71]. For example, brain tumor glioblastoma multiforme cells are highly sensitive to ECM stiffness but their cytoskeletal stiffness is irrespective of ECM stiffness when talin-1 is depleted [72]. Moreover, inhibiting the paxillin–vinculin interaction or depleting vinculin reduces FA force transmission and depletes tugging FA traction dynamics [73]. Above the ‘force transduction layer’, the ‘actin regulatory layer’ is situated, in which proteins like α-actinin, zyxin, and vasodilator-stimulated phosphoprotein (VASP) accumulate and induce actin nucleation and polymerization to modulate the cytoskeleton. Of these proteins, zyxin facilitates cytoskeletal tension-dependent actin polymerization at FAs [74] and α-actinin integrates mechanical forces to establish actin network symmetry [75]. Collectively, these studies highlight the complex mechanosensitive mechanisms that enable stiffness-sensing at the FA-ECM border.

### 5.2. FA Focal Complex

The starting point of a FA is called a focal complex, which is built at the lamellipodia of cells and regulated by Rac1 and Cdc42. A focal complex is characterized by its high throughput assembly of proteins. Upon mechanical stimulation, the focal complex matures to a FA and F-actin is assembled and cross-linked by myosin phosphatase II (myosin II) [76]. The focal complex becomes a mature FA and it has been shown that force input induces growth and FA maturation [77]. On substrates with increased stiffness, more integrins aggregate, which leads to enlarged FA complexes with enhanced aggregation of proteins and polymerization of more actin fibers. Spreading cells use many lamellipodia, which then establish new FA complexes [53,78].

### 5.3. Integrins

In this context, integrins are FA key elements. This transmembrane receptor family consists of 18 α- and 8 β-subunits, building many heterodimers. The extracellular domain of integrins allows them to recognize ECM proteins such as fibronectin [79], collagen [80], laminin [81] as well as other ECM proteins. The cytoplasmic tail of the integrins enables interactions with various FA proteins. The β-subunit binds to proteins like talin, which binds to the cytoplasmic tail of integrins [82], to α-actinin, an actin filament crosslinking protein [83], and to kindlin, which is also a regulator of integrin activation and cytoskeletal reorganization [84], as integrins cannot directly bind to the actin cytoskeleton [85,86,87].

### 5.4. Focal Adhesion Kinase

**Figure 2:**
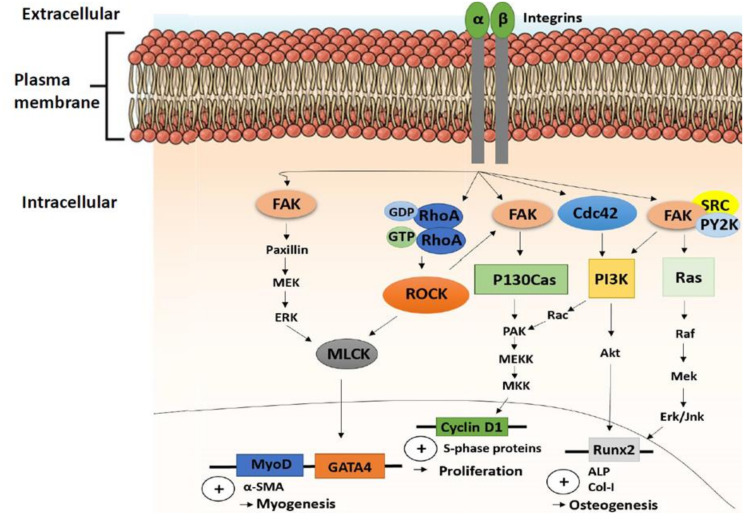
Stiffness-induced integrin-dependent focal adhesion kinase (FAK) signaling. Integrin stimulation leads to an activation of various downstream effectors. One of them is FAK, which binds Src and Pyk2 and activates Ras, which further activates, Raf, MEK and extracellular signal-regulated kinase (ERK)/Jun NH2-terminal kinase (JNK). Ultimately leading to expression of runt related transcription factor 2 (RUNX2) and osteogenic differentiation of mesenchymal stromal cells (MSCs). FAK can also activate phosphoinositide 3-kinase (PI3K), which stimulates Akt and also increases expression of RUNX2. Integrins can also activate Cdc42, which activates PI3K. This leads to activation of Rac, p21-activated protein kinase (PAK), MEK kinase-1 (MEKK), MAP kinase kinase (MKK) and expression of cyclin D1 to enhance cell proliferation. Another effector of FAK is P130Cas, which also stimulates expression of cyclin D1. Integrins also activate RhoA, which binds guanosine-5’-triphosphate (GTP) and activates RhoA/Rho associated protein kinase (ROCK). ROCK activates myosin light chain kinase (MLCK) to eventually increase expression of MyoD and therefore myogenic differentiation of MSCs. FAK can also activate paxillin, which leads to further downstream signaling through MEK, phosphorylation of ERK and MLCK. MLCK then leads to enhance actin-myosin expression and myogenic differentiation.

FAK is a central element in mechanotransduction, as it is involved in both inside-out and outside-in signaling activation [88,89,90,91] and, thus, controls endogenous cytoskeleton contractility and multiple other cell functions (Figure 2). The recruitment of FAs leads to activation of FAK through phosphorylation [92], which then participates in actin polymerization and, thus, is generally involved in cellular adhesion, proliferation, and cell spreading. Cell proliferation is induced through activation of extracellular signal-regulated kinases (ERKs) via FAK. In addition, cell migration is controlled by FAK, which sequesters and activates Src family kinases, leading to further phosphorylation of p130 Cas and Rac1 [93]. Another target of activated FAK is paxillin, which initiates mitogen-activated protein kinase kinase (MEK), leading to downstream activation of ERK1/2 and the myosin light chain kinase (MLCK) to control endogenous actin contractility of the cytoskeleton. ERK1/2 controls the differentiation of stem cells into osteocytes on rigid substrates [94] and is involved in the differentiation of cardiac fibroblasts to myofibroblasts in a stiffness-dependent manner [95]. In addition to differentiation control, ERK1/2 mediates cell proliferation and apoptosis [96,97]. ERK1/2 is also regulated by the Rho protein kinases Rac, Rho, and RhoA/Rho associated protein kinase (ROCK) [94,98,99,100]. Other studies have shown that the RhoA/ROCK pathway is also capable of influencing myosin contractility and is activated through FAK and Src [101,102,103].

### 5.5. Rho GTPases 

The family of Rho GTPases includes 20 protein members and the most prominent ones involved in stiffness sensing are RhoA, Rac1, and Cdc42. Biomechanical tension activates RhoA/Rho associated protein kinase (ROCK) signaling, which implies that matrix stiffness also modulates cell cytoskeletal organization. Indeed, on hard substrates, an increase of RhoA expression and its contributing effect on the formation of actin stress fibers, compared to less stiff substrates, was demonstrated [104]. The RhoA/ROCK pathway also influences myosin contractility and is activated through FAK and Src [101,102,103]. RhoA is also activated by guanine-exchange factors (GEFs) and, just as RhoA [105], demonstrated that under mechanical force input GEF-H1 shows an increased activity. Activated RhoA increases actin nucleation and polymerization to induce formation of stress fibers [104] via the diaphanous formins, mDia1 and mDia2, leading to long and straight actin fibers [106]. ROCK is a serine/threonine kinase and a downstream effector of active RhoA. Through phosphorylation of the myosin-binding subunit of myosin II, cross-linking of actin filaments is initiated. Another target of ROCK is LIM kinase-1 (LIMK1). Phosphorylated LIMK1 leads to phosphorylation and therefore inactivation of cofilin, which normally inhibits actin polymerization. ROCK activity increases with substrate stiffness, as cells on stiffer substrates have a higher ROCK activity than cells cultured on softer substrates [107]. To demonstrate that stiffness sensing on soft substrates also occurs in CHs through ROCK, one study treated primary murine CHs (mCHs) with the ROCK inhibitor Y27632 and demonstrated a complete repression of the ROCK-dependent expression of collagen type II and SRY-related HMG box-containing (SOX9) [108], an early chondrogenic gene marker. This confirmed that ROCK plays a key role in the stiffness sensing ability of (m)CHs ^108^. To sum it up, the RhoA/ROCK signaling pathway establishes a functional actin cytoskeleton and studies have shown that biomechanical stiffness changes expression of RhoA and concomitantly ROCK [104]. Moreover, there is extensive crosstalk between integrins, Src-family kinases such as FAK and the Rho-family GTPases at the center of adhesion signaling [109].

### 5.6. Stress Fibers

Stress fibers are bundles of contractile F-actin filaments, which are mainly cross-linked by non-muscle myosin II. Four types of stress fibers have been identified: dorsal stress fibers, ventral stress fibers, transverse arcs, and the perinuclear actin cap, which is a mediator of nuclear mechanotransduction [110]. However, this has not yet been demonstrated, as no F-actin structural sub-analysis has been performed in CHs. The filamentous singular units are monomeric G-actin and filamentation is initiated by the formin-family of actin nucleators/elongation factors, like filamin, α-actinin, and cortactin, which are activated by the Rho GTPases and phosphoinositides [111,112,113]. The branched filaments are formed through actin-related protein 2/actin-related protein 3 (Arp2/3) and other nucleation promoting factors. Branched actin is then formed at the site of Arp2/3 from the existing actin filament [114,115] and cross-linked by myosin II. Interestingly, stiffer substrates increase intracellular contractility through an increase in stress fibers [115].

## 6. Material Stiffness-Regulated Cell Proliferation

ECM stiffness is able to regulate cell proliferation. Studies that increased substrate stiffness from softer to stiffer also increased the proliferation of cancer cells, fibroblasts [116,117,118,119,120], as well as human MSCs (hMSCs) [121] and rat CHs (rCHs) [122]. In this context, Rho GTPases, especially Rac1, are involved in modulating the expression of cyclin D1, which promotes S-phase entry of cells needed for inducing proliferation [39]. Additionally, proliferation can also be initiated through integrin clustering. Subsequently, recruited FAK autophosphorylates bind Src and activate p130Cas. Further downstream, either Jun NH2-terminal kinase (JNK) or Rac are then activated. Phosphorylated JNK leads to increased expression of cyclin D1 [123,124]. Rac1, which has been shown to be involved in FAK and p130Cas signaling [39], is required for induction of cyclin D1 [125]. Thus, molecular pathways involved in stiffness sensing modulate S-phase entry and control the proliferation of cells. Interestingly, proliferation can also be regulated through the transcription co-activator of the yes-associated protein (YAP) and its transcriptional co-activator with PDZ-binding motif (TAZ) [126], as cells grown on stiff substrates build more F-actin, spread, and display active YAP/TAZ in the nucleus. In turn, active nuclear YAP/TAZ promotes the proliferation of multiple cell types [127]. Cells cultured on relatively soft substrates accumulate YAP/TAZ in the cytoplasm and thus, show a reduction of their proliferation rate [126,128]. Accordingly, on soft substrates, YAP retained in the cytoplasm undergoes a degradation process [129], whereas stiff substrates induce YAP to translocate into the nucleus [130], due to contractile forces generated by actomyosin activity that flatten the nucleus and open up nuclear pores. However, in hMSCs active nuclear YAP/TAZ appears to have no role in proliferation but promotes osteogenesis [121] and the effects of YAP to promote osteogenic differentiation is based on an interaction of YAP with β-catenin [131]. In the context of endochondral ossification, proliferation of early committed CHs is increased with YAP expression [132]. In AC rCHs YAP downregulation on soft substrates helps maintain the CH phenotype while inhibiting CH proliferation [122]. Collectively, it has been demonstrated that harder substrates induce relatively more cell spreading and proliferation than softer substrates [133,134,135], and increased adhesion and traction forces. This was shown to be also true for hMSCs [121] and rCHs [122]. Such a phenomenon correlate with the amount of active RhoA expression [134,136], as cells on softer substrates exhibit less spreading and reduced proliferation and FA assembly.

## 7. Material Stiffness-Regulated Cell Migration

Cells can sense substrate stiffness through probing and contraction of actin fibers and migrate towards substrate areas of higher stiffness [137,138]. This movement is explained by the molecular clutch hypothesis. At the leading edge of the lamellipodium, new actin monomers are incorporated into the rising actin filament. Integrins are bound to the ECM upon activation and cluster. Integrin signaling promotes actin polymerization and matures FAs, which then are composed of a number of proteins that connect the ECM and the cytoskeleton. Force is transmitted to the ECM and the lamellipodium becomes the leading edge of the cell. On softer substrates, lamellipodia are unstable and integrins are not engaged by the ECM, leading to less actin polymerization, rapid retrograde cytoskeletal flow, and no net protrusion. Thus, traction forces from the cell are not transmitted to the ECM and, consequently, the cell does not move. Depending on the magnitude of the elastic modulus, hMSCs migrate faster on softer substrates such as 3 kPa and form smaller FAs, compared to a slower movement on substrates with a higher elastic modulus such as 30 and 600 kPa [139]. However, on gradients within the range of physiologically relevant elastic moduli for soft tissues (i.e., 1–12 kPa), hMSCs migrated to the stiffest region on each gradient and their migration speed correlated with the gradient strength [140]. Thus, material stiffness controls the direction and speed of hMSC migration on stiffness gradients.

## 8. Material Stiffness-Modulated MSC Shape and Lineage Determination

Material stiffness controls many cell functions such as cell shape [42], adhesion [141], migration [137], differentiation [44], and proliferation [142,143,144]. Recently, cell morphology has received re-ignited attention, as measuring, predicting, and controlling cellular shape may aid in future regenerative medicine applications [145]. The morphology of MSCs is influenced by microenvironmental and biophysical stimuli [44,45,136,146,147] and is defined by how the cell balances external biomechanical forces with intracellular forces. The level of internal forces is directly proportional to the biomaterial stiffness of the substrate [45]. In one of our studies [148], the shape of hMSCs was engineered using (i) different biomaterials with similar stiffnesses vs. using (ii) the same biomaterial materials with different stiffnesses. Indeed, higher nanoscale stiffness, compared to a lower stiffness of the same biomaterial, was associated with rounder hMSCs, high aspect ratio and circularity, and a lower solidity. Thus, hMSCs cultured on biomaterials with different stiffnesses adopted cell shapes, which are characteristic of the used biomaterial. Interestingly, when comparing the effects of biomaterial stiffness vs. cyclic tension on hMSC shape [148], dynamic tensile forces were more effective in defining hMSC shape than substrate stiffness. However, the biomechanical effects on cell shape were transient; once the application of mechanical force had been stopped, hMSC shape ultimately reversed back to the shape dictated by substrate stiffness. In accordance to stiffness-defined alterations in the shape of MSCs, hMSCs committed to a neuronal cell type lineage on soft hydrogels and adopted the dendritic, neuron-specific cell shape, whereas hMSCs underwent osteoblast differentiation on rigid substrates and adapted the polygonal [44]/cuboidal [149] osteoblast-specific shape. Thus, material stiffness modulates both MSC morphology and accompanying lineage determination.

Since the stem cell ability of self-renewal and differentiation potential makes MSCs especially attractive for applications in regenerative medicine [150], it is relevant to further explore MSC characteristics and potential therapeutic usage in the context of material stiffness. For MSCs in particular, it has been shown that matrix stiffness has a major influence on hMSCs lineage determination [44]. This study was the first to demonstrate in a 2D system that, in the absence of exogenous soluble factors, hMSCs can be differentiated in vitro into specific tissue lineages, and that this lineage commitment depended on substrate stiffness. Moreover, the cell fate-deciding stiffness corresponded to the in vivo mechanical tissue stiffnesses. Interestingly cell adjustment to the microenvironmental material properties relied on non-muscle myosin II, together with alterations in adhesion mechanics and the actin cytoskeleton structure [44]. In this context, multiple studies that used 2D systems confirmed that substrate stiffness controls the differentiation potential of MSCs. Cells grown on soft substrates differentiate towards the neurogenic lineage, whereas intermediate stiffnesses induce myogenic differentiation, and higher stiffnesses commit MSCs to an osteogenic fate [94,151,152,153,154]. Some of these fate-dependent decisions may be controlled through the YAP transcription factor as we explained above. For example, adipo-osteogenic differentiation of hMSCs has been shown to be regulated in part by YAP [155]. In this context substrate mechanics control adipogenesis through YAP phosphorylation by dictating cell spreading [156]. Additionally, YAP is a negative regulator of chondrogenic differentiation of MSCs, as downregulation of YAP for chondrogenesis is needed to alleviate the repressing effect of nuclear YAP on chondrogenic signaling [157].

Like MSCs, human adipose-derived stem cells (hASCs) and neural stem cells (NSCs) are also influenced by substrate stiffness. On substrates with a stiffness similar to in vivo adipose tissue, hASCs differentiate into adipocytes, whereas on substrates with a stiffness complementary to muscle tissue the cells undergo myogenic differentiation and are capable of building myotubes [158]. For rat NSCs (rNSCs), soft substrates promote neurogenesis, whereas rNSCs on harder substrates differentiate into oligodendrocytes [159,160]. Together, these studies highlight lineage determination of stem cells by material stiffness.

## 9. Material Stiffness-Modulated CH Shape, Cytoskeleton, and Phenotype

Biomaterial stiffness might be usable as a potential regeneration-inducing determinant, as the previous text section of this review discussed how material stiffness in the context of MSCs controls cell morphology and associated cell function(s). Thus, it would be beneficial to better understand how substrate stiffness influences the behavior of healthy and diseased CHs, and how such improved insight might be translated into improved strategies for AC repair strategies. Moreover, it has been well-established that a critical decrease in ECM stiffness has been implicated in OA-related changes in CH phenotype [14], which illustrates that material stiffness is not only a parameter relevant for CH culture but also an important aspect of the many OA pathomechanism(s).

ECM mechanical cues including ECM stiffness, cell attachment or detachment, and cellular tension are potent regulators of YAP/TAZ [161]. A critical decrease in ECM stiffness has been implicated in OA-related changes in CH phenotype [14]. In fibroblasts, ECM stiffness mechanoactivates YAP/TAZ, which promote the production of pro-fibrotic mediators and ECM proteins. This results in tissue stiffness-mediated YAP/TAZ signaling as a molecular link between fibrosis and cancer [161] and illustrates how stiff substrates can contribute to inducing fibrotic changes. However, in AC, OA-associated AC degradation is in part regulated by a reciprocal inhibition of YAP/TAZ and NF-κB (nuclear factor ’kappa-light-chain-enhancer’ of activated B-cells) signaling [162], which illustrates a potential material stiffness-mediated role of YAP/TAZ in OA AC degradation. To clarify, YAP inactivation is conducive to the maintenance of a chondrogenic phenotype [122], because relatively stiff substrates (40 kPa) increase YAP expression and YAP accumulation in the nucleus of rCHs, concomitant with high expression levels of collagen I and almost no collagen type II expression. In turn, relatively soft substrates (4 kPa) decrease YAP expression and cytoplasmic YAP accumulation, concomitant with high expression levels of collagen type II, SOX9, and aggrecan (ACAN). Additionally, YAP knockdown of rCHs on stiff substrates displayed significantly increased collagen type II, SOX9, and ACAN and decreased collagen type I expression.

An extensive study that focused on mechanistic aspects cultured mCHs in 2D on type II collagen-coated polyacrylamide (PAA) gels with elastic moduli between 4 and 31 kPa with a constant adhesion ligand composition [163]. In 2D, increasing the elastic modulus induced mCH catabolism, downregulation of AC ECM molecules, disrupted SOX9 nuclear localization, and decreased SOX9 transcriptional activity. Softer 2D substrates (≤7 kPa) induced a round mCH morphology and stiffer substrates (12–31 kPa) promoted FAs and stress fiber formation. In mCHs on stiff substrates, Rho and ROCK activities were increased and the inhibition of Rho with C3 transferase, of ROCK with Y27632, and of myosin II ATPase with blebbistatin, or disruption of F-actin with cytochalasin D abolished stiffening-mediated FA and stress fiber formation, upregulation of matrix-degrading enzymes, downregulation of collagen type II (COL2A1), ACAN, and SOX9, and inhibition of SOX9 activity by restoring SOX9 nuclear localization [163]. The here discussed study also used 3D collagen matrices for increasing the elastic modulus with lysyl oxidase (LOX), also known as protein-lysine 6-oxidase, which catalyzes the conversion of lysine molecules into reactive aldehydes, which form cross-links in ECM proteins. The LOX-treatment increased the 3D collagen hydrogel elastic modulus from <65 Pa to 90 Pa and induced in the embedded CHs an increase in the mRNA expression of matrix metalloproteinase (MMP)-3, MMP-13, and a disintegrin and metalloproteinase with thrombospondin motifs 5 (ADAMTS5), and a decrease in collagen type II and ACAN expression [163], indicating than increasing the elastic modulus in 3D has comparable effects on hCHs in 3D vs. 2D.

In the context of the role of the elastic modulus-modulated CH phenotype, one study cultivated porcine CHs (pCHs) for two weeks in 3D agarose hydrogels with different substrate elasticities (3.7 vs. 53.2 kPa) and protein-modulated adhesion site densities [164]. Interestingly, the pCHs maintained their chondrogenic phenotype independently of the substrates, but softer gels led to higher DNA and glycosaminoglycan (GAG) contents and larger cell clusters than stiff gels. Since this occurred in both Arg-Gly-Asp (RGD)- and arginine-glycine-glutamic acid (RGE)-modified agarose, the authors hypothesized that matrix elasticity in the tested range did not influence the maintenance of the chondrogenic phenotype in 3D but rather the size of the formed cell clusters [164]. However, another study explained such findings differently and suggested cell sensing of cell volume confinement as an adhesion-independent mechanism of mechanotransduction in 3D culture [165]. Whether such a mechanism is subject to substrate stiffness has not been demonstrated yet. Another study investigated how matrix elasticity influences CH differentiation and phenotype. pCHs were cultured for seven days in 2D on polyacrylamide (PAA) hydrogels having lower (4 kPa) and higher elastic moduli (10, 40, and 100 kPa) [166]. Interestingly, pCHs on 4 kPa PAA hydrogels maintained a CH phenotype, as indicated by a higher expression of collagen type II, ACAN, and lower expression of collagen type I. pCHs did not proliferate and exhibited a diffuse actin organization with round cell morphology. On hydrogels with higher elastic moduli (10, 40, and 100 kPa) the cells displayed spread morphology, organized actin fibers, and higher proliferation rates. With increasing elastic modulus, the gene expression of collagen type II decreased, whereas the expression of ACAN and collagen type I increased. Another study [167] demonstrated that culturing hCHs in 2D on 300 g/mol poly(ethylene)glycol (PEG) substrates led to a spread morphology with distinct stress fibers, whereas culturing on 1000 g/mol PEG substrates led to cells having a round morphology, a cortical actin structure, and protein kinase C expression [167]. Here, increasing the molecular weight or decreasing the concentration of PEG reduced the crosslinking density, which resulted in a softer hydrogel [168]. Another study that investigated the behavior of mCHs on different polydimethylsiloxane (PDMS) stiffness substrates quantitatively with atomic force microscopy (AFM) demonstrated that a stiffer substrate tended to increase the cell spreading area and the percentages of irregular, fibroblast-like cell shapes as well as increased mechanical parameters such as elastic modulus, instantaneous modulus, relaxed modulus, and the viscosity of mCHs [169].

In summary, a few studies demonstrated that material stiffness controls CH proliferation, morphology, phenotype, and mechanical characteristics. It is noteworthy that the used culture systems differed greatly in their stiffness values, making comparisons difficult. However, one can conclude that softer substrates foster a more chondrogenic phenotype than harder ones, and that 2D systems with an elastic modulus value of approximately 4 kPa but not ≥10 kPa appear suitable for inducing or stabilizing a chondrogenic phenotype in CHs. However, in 3D the elastic modulus values (e.g., for CHs GAG accumulation) appear much lower. Mechanistically, increasing the elastic modulus promotes FA and stress fiber formation and CH catabolism, which have been associated with the Rho-ROCK-MLC pathway (MLC: myosin light chain). In this context, ROCK and RhoA have been shown in another study to be key modulators of actin cytoskeleton tension and FA formation [170]. Moreover, a study demonstrated an inverse correlation between cCH differentiation and the level of activated (GTP-bound) RhoA [171].

## 10. Material Stiffness Changes Modulate Nuclear Shape and Nuclear Lamina and Inner Membrane Composition for Controlling mRNA Expression and MSC Differentiation

The cytoskeleton is mechanically linked to the nucleus by the linker of nucleoskeleton to cytoskeleton (LINC) complex, which consists of nuclear envelope embedded proteins [172]. Key components of the LINC complex are lamins, which are class V intermediate filament family proteins that form the nuclear lamina under the inner nuclear membrane. Lamins occur in types, namely, A- and B-types, whereas the C-type is an isoform of A [172]. Interestingly, it has been demonstrated that MSC differentiation into adipose tissue on soft matrix was enhanced by low lamin-A levels, whereas osteogenic differentiation on stiff matrix was enhanced by high lamin-A levels [173]. Moreover, induced lamin-A overexpression in combination with stiff matrix and inducing media favored MSC osteogenesis [173]. These data can be explained by evidence that suggests mRNA expression is mediated by nuclear morphology as demonstrated by previous research [174], mediated in part by a link between the nucleoskeleton and the cytoskeleton at the nuclear envelope that provides a mechanism for transmission of mechanical forces into the nucleus [175]. Additionally, the nuclear shape is modulated by substrate rigidity-induced changes in the actomyosin tension and, thus, a mechanically integrated nucleus-cytoskeleton is required for material stiffness sensing [174]. In the context of this review, it is helpful to know that both A-type lamins and transcriptionally active chromatins are vertically polarized by the tension exercised by the perinuclear actin cap (or actin cap) [176], which is a specific type of stress fiber of the cytoskeleton that is linked to the nucleus via LINC complex [172]. This mechanical link illustrates how extracellular biophysical cues such as material stiffness impact on cell behavior via modulating the ECM–FA–cytoskeleton–actin cap–nucleus axis. In the context of material stiffness, this axis has been connected to MSC osteogenesis [173] but not yet to CH phenotype.

## 11. TGF-β1-Induced Lineage Determination of MSCs is Modulated by Material Stiffness

MSCs are not only controlled by substrate stiffness, but by many other factors, including growth factors. One well-understood example is transforming growth factor β (TGF-β), which can also modulate MSC lineage differentiation. One study investigated the effect of TGF-β1 on hMSC differentiation into either smooth muscle cells (SMCs) or CHs, when cultivated on substrates with different stiffnesses [121]. They demonstrated that the stiffness of the cell adhesion substrate modulated the effect of TGF-β1, as hMSCs on soft substrates spread less, showed fewer stress fibers, and lower proliferation rates, compared to hMSCs on stiff substrates. Moreover, hMSCs differentiated on softer substrates into the chondrogenic lineage and on substrates with intermediate stiffness into the myogenic lineage. Constitutively activated RhoA in hMSCs increased the expression of smooth muscle cell (SMC) marker genes on stiff substrates but collagen type II and lipoprotein lipase (LPL) on soft substrates, which suggested material stiffness-specific mRNA upregulation of chondrogenic and adipogenic genes through RhoA [121].

Interestingly, in synovium-derived mesenchymal stem cells (sMSCs) on plastic, incubation with TGF-β1 induced RhoA activity and ROCK1 and 2 expression, which gradually decreased after four days. Additionally, the TGF-β1-stimulated cells showed a dramatically increased cytoplasmic stress fiber staining and chondrogenic RNA expression [177]. When RhoA/ROCK inhibitors were added, the TGF-β1-induced cytoskeletal reorganization was interrupted, and chondrocyte-specific genes were downregulated [177].

## 12. TGF-β1- and IL-1 β-Induced Changes in CH Stiffness and Traction Force are Material-Stiffness Dependent

**Figure 3:**
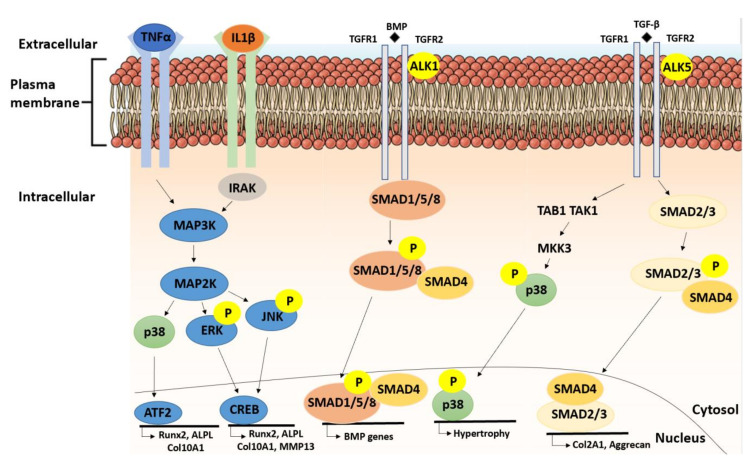
Tumor necrosis factor beta (TNFα), interleukin 1 beta (IL-1β), bone morphogenetic protein (BMP), and transforming growth factor beta (TGF-β) are stiffness-dependent signaling pathways. Binding of TNFα to its receptor leads to activation of the mitogen-activated protein kinase (MAPK) pathway. MAP3K and MAP2K are activated and three groups of MAP kinases are involved: ERK, JNK, and p38 MAP kinase. Activation of p38 further activates transcription factor activation transcription factor 2 (ATF2), which enhances transcription of osteogenic genes such as RUNX2, liver/bone/kidney alkaline phosphatase (ALPL) and hypertrophic collagen Col10A1. Phosphorylation of ERK and JNK leads to activation of cAMP response element-binding protein (CREB) and also the activation of RUNX2, ALPL, Col10A1, and additionally matrix metalloproteinase 13 (MMP-13). The binding of IL-1β activates interleukin-1 receptor associated kinase (IRAK) and leads to similar downstream effectors in chondrocytes (CHs). BMP signaling occurs via the SMA- and MAD-related protein (SMAD)-dependent pathway. Signaling is initiated by binding to type I or type II serine/threonine kinase receptors and forming a heterotetrameric complex. The type I receptor is transphosphorylated by constitutively active type II receptor and activin receptor-like kinase 1 (ALK1), which activates R-SMAD1/5/8. Phosphorylated R-SMAD1/5/8 then binds to its co-receptor SMAD4 and translocates into the nucleus, where it initiates transcription of BMP-specific genes (e.g., RUNX2). TGF-β is initiated by ligand binding to receptor types I and II with ALK5. SMAD2/3 gets activated and also binds to co-activator SMAD4. The translocation into the nucleus activates transcription of chondrogenic genes like collagen type II and aggrecan (ACAN). Ligand binding also activates mitogen-activated protein kinase kinase kinase 7 (MAP3K7, also known as TAK1) and TGF-β activated kinase 1 (MAP3K7) binding protein 1 (TAB1), leading to further activation of MKK3 and phosphorylation of p38. Phosphorylated p38 leads to transcription of RUNX2 and MMP-13.

One study examined how ECM stiffness affects the response to the chondrogenic growth factor TGF-β, an agonist of CH differentiation, and how ECM stiffness affects mechanosensitive TGF-β1 expression [108]. The authors cultured mCHs and ATDC5 cells (a cell line derived from mouse teratocarcinoma cells) on PAA hydrogels with different elastic moduli (0.2, 0.5, and 1.1 MPa). They demonstrated that the expression levels of SOX9, collagen type II, ACAN, and endogenous TGF-β were highest on 0.5 MPa substrates in mCHs, whereas the response to the chondrogenic growth factor TGF-β measured in ATDC5 cells was higher on 0.5 MPa vs. plastic substrates. Interestingly, the study also induced the expression of collagen type II in mCHs on 1.1 MPa hydrogels by using ROCK inhibition, illustrating the stiffness-dependent effect of the mCH cytoskeleton on mCH phenotype. The authors suggested a synergistic response of TGF-β and substrate stiffness and also demonstrated that this response was dependent on p38 mitogen-activated protein kinase (MAPK) signaling rather than SMAD3 [108]. Another study that evaluated the effects of stiffness on CHs cultivated goat CHs (gCHs) on PAA hydrogels with substrate elastic moduli of 1, 11, and 90 kPa and demonstrated that increased stiffness led to increased gCH actin stress fibers and FAs [178]. Moreover, the study demonstrated that TGF-β1 increased cellular stiffness and traction force, while IL-1β increased cellular stiffness but lowered traction force. Interestingly, the TGF-β1 effects were potent on 90 kPa substrates and IL-1β effects on 1 kPa substrates [178]. Although this study did not elucidate on mechanistic details, it is conceivable that the findings can be explained in part by increases in actin polymerization because it has been demonstrated that TGF-β1 treatment of synovium-derived rMSCs leads to increased F-actin stress fiber formation [177]. Furthermore, TGF-β1 is known to induce cell stiffening in bovine CHs (bCHs) and it has been proposed that this stiffening is based on a combination of integrin activation from cellular attachment and increased actin polymerization from stimulation with TGF-β1 (and IGF-I) and subsequent increases in F-actin [179]. Another more recent study confirmed that rabbit CHs (rabCHs) treated with TGF-β1 show enhanced F-actin [180]. Thus, that TGF-β1 increases cellular stiffness and traction force as previously reported [178] for gCHs can be explained by TGF-β1 increasing F-actin stress fiber formation. Mechanistically, TGF-β-induced actin reorganization appears to be mediated by Smad proteins and Rho GTPases, as demonstrated in Swiss 3T3 fibroblasts [181]. A potential explanation for the IL-1β effects on increased gCH stiffness as reported prior [178] can be derived from a study, which observed a disassembled appearance of actin, tubulin, vimentin, and vinculin in both healthy and OA hCHs after IL-1β stimulation [182], as vimentin forms a tight, interconnected inner network that contributes to cytoskeletal stiffness [183]. The effects of IL-1β on increased cell stiffness can also be explained by another study that reported increased stress fiber formation after IL-1β treatment [184]. The effects of IL-1β on lowered traction force as reported previously [178] can be explained by effects on multiple mechanotransducing proteins, as IL-1β is able to decrease the expression of tensin, talin, paxillin, and FAK in mCHs in an actin polymerization-dependent fashion [185], as inhibiting the paxillin–vinculin interaction or depleting vinculin reduces FA force transmission and depletes tugging FA traction dynamics [73]. Thus, growth factor- and pro-inflammatory cytokine-induced changes in cellular stiffness and traction force are material-stiffness dependent. The subsequent signaling is illustrated in Figure 3.

## 13. Substrate Stiffness-Modulated Cell Surface Growth Factor Receptor Composition

One study demonstrated that TGF-β receptors (TβR) are discretely organized to segregated spatial domains at the cell surface, and that disruption of cellular tension leads to a collapse of this spatial organization, which, in turn, drives formation of heteromeric TβRI/TβRII complexes and Smad activation [186]. Thus, this study elucidated a novel mechanism by which cellular tension regulates TGF-β receptor organization and function, which helps to explain the observation reported by Park et al. [121] that TGF-β1-induced lineage determination of MSCs is modulated by material stiffness. Substrate stiffness was also shown to influence the cell surface receptor composition in rat MSCs (rMSCs) [187]. On soft substrates the bone morphogenetic protein (BMP) type I receptor, which complexes with β1 integrin, undergoes increasing activation, and is internalized through a caveolae/raft-dependent endocytosis. This internalization repressed the BMP/Smad pathway at least partially through integrin-regulated BMP receptor endocytosis, blocking the neural lineage specification of rMSCs on soft substrate. Moreover, the study suggested that ECM elasticity affects integrin activity and trafficking to modulate integrin BMP receptor internalization, which, in turn, contributes to stem cell lineage specification [187]. CHs generate an integrated response to ECM stiffness and transforming growth factor β (TGF-β) [108] that can be compared to the TGF-β1-induced lineage determination of MSCs described by Park et al. [121]. However, stiffness-modulated effects of CH cellular tension on the TGF-β receptor organization and function in CHs have not yet been described.

## 14. Rho GTPases in Substrate Stiffness-Modulated MSC Differentiation and CH Phenotype

As demonstrated by Park et al. [121], substrate stiffness modulates the effects of TGF-β1 on hMSC myogenic vs. chondrogenic differentiation fate. Interestingly, Rho GTPases, RhoA activity, Rho-induced stress fiber formation, and α-actin assembly were the deciding factors in lineage determination. In this context, the data in Park et al. [121] suggested that larger amounts of activated RhoA were present in hMSCs on stiffer than softer substrates. To further elucidate, whether RhoA regulates differential gene expression, the group overexpressed constitutively active RhoA in hMSCs. RhoA activation significantly increased expression of SMC marker genes on stiff substrates but collagen type II and LPL on soft substrates, which suggested stiffness-specific mRNA upregulation of chondrogenic and adipogenic genes through RhoA [121]. Mechanistically, another study suggested in this context that spread cells contain similar amounts of total ROCK (a kinase and downstream effector of active RhoA) comparable to round cells but higher amounts of activated ROCK and more pronounced stress fiber formation, when cells underwent osteogenic differentiation [136]. Another study [188] highlighted that chondrogenic or myogenic hMSC lineage determination was dependent on cell shape, Rac1, and N-cadherin. Through dose-dependent activation of Rac1, the fate decision of the hMSCs was controlled on compliant adhesion sites [188].

In 2010, Haudenschild et al. [189] found a consensus phosphorylation site in SOX9 for ROCK, which directly links SOX9 transcriptional activity to a ROCK–SOX9 interaction. The authors demonstrated that ROCK phosphorylates SOX9 at Ser181, which increases nuclear accumulation of SOX9 protein (e.g., in response to mechanical compression and TGF-β1) [189]. Indeed, two other 2D studies demonstrated that the modulation of the RhoA/ROCK pathway controls the transcription of SOX9 for promoting chondrogenic differentiation [189,190]. At first glance, these two studies appeared to report conflicting data, as pharmacologically inhibiting ROCK, a downstream effector of active RhoA, resulted in elevated SOX9 expression levels in ATDC5 cells [190], whereas Haudenschild et al. [189] reported that increasing amounts of ROCK show a dose-dependent increase in SOX9 transcriptional activity in hCHs. However, this apparent conflict can be resolved by material stiffness-specific effects of ROCK, as demonstrated by Allen, Cooke, and Alliston [108]. In that study, high SOX9 expression levels were quantified in ATDC5 cells when cells were cultured on chondrogenic elastic modulus levels of 0.5 MPa and low SOX9 expression levels on plastic culture dishes. The pharmacological inhibition of ROCK resulting in elevated SOX9 expression as reported in a previous study [190] was observed only on plastic culture dishes, whereas ROCK inhibition of cells on chondrogenic stiffness levels resulted in decreased SOX9 expression levels, illustrating an interesting material stiffness-specific effect of ROCK on SOX9 expression. Thus, the data illustrate that higher stiffness does lead to higher SOX9 but also that higher SOX9 does not necessarily lead to increased chondrogenic gene expression. Thus, in a 2D situation, chondrogenic elastic modulus such as 0.5 MPa as reported in one study [190] leads to SOX9 levels that act chondrogenically. Higher SOX9 levels that occur in cells cultured on higher stiffness such as on plastic act non-chondrogenically. In line with this explanation, another study demonstrated in chicken CHs (cCHs) on 2D plastic dishes an inverse correlation between CH differentiation and the level of activated (GTP-bound) RhoA [171]. This inverse correlation has also been observed in the same study in cCHs in 3D alginate gel culture and in limb bud mesenchymal cell micromass culture, but 2D vs. 3D systems cannot be directly compared. In this context, a direct modulation of ROCK activity through material stiffness was reported by Huang et al. [104], in which a stiffer matrix promoted increased RhoA production and also increased the activation of RhoA in the membrane but not in the cytosolic fraction, followed by subsequently increased ROCK activity on a stiffer matrix.

In summary, the roles of the Rho GTPases and of RhoA/ROCK in particular in modulating CH phenotype are not sufficiently understood. On the one hand, the ROCK–SOX9 interaction through a consensus site serves well for explaining the effects of ROCK on CH phenotype, as SOX9 is a transcription factor essential for the formation of all cartilaginous tissue [191]. In this context, RhoA/ROCK signaling acts pro-chondrogenic. On the other hand, it has been demonstrated that ROCK induces stress fiber formation by phosphorylating MLC [192,193]. Other studies specified that activated RhoA increases actin polymerization to induce stress fiber formation [104], and that ROCK inhibition supports the establishment of a CH-specific cell shape and actin organization [190]. Interestingly, cytochalasin D, an inhibitor of actin polymerization, can reverse the de-differentiated phenotype of monolayer-passaged CHs [194] but the subsequent mechanisms of how a chondrogenic CH phenotype is being restored are less clear. In this context, RhoA/ROCK signaling acts anti-chondrogenically and induces CH de-differentiation. How material stiffness through modulation of ROCK activity impacts this apparent balance between the pro- and anti-chondrogenic effects of RhoA/ROCK signaling remains unclear.

## 15. Substrate Stiffness-Modulated Integrin Subunit Expression of MSCs and CHs

Integrins are an integral part of FAs. A differential integrin expression regulated by substrate stiffness has been noted in MSCs and CHs. For example, the expression of integrin α1, α2, and α5 has been reported to be much more sensitive to stiffness in hMSCs than in human osteoblasts and hCHs [195]. Another study [196] investigated the expression of cell surface integrins in rCHs on hydrogels with elastic moduli of 2, 10, and 20 Pa under normoxia vs. hypoxia. Blocking various integrin subunits and assessing subsequent aggrecan (ACAN) expression, the authors concluded that the integrins α1, β1, αVβ3, and β3 were involved in mechanosensing, whereas the integrins α2, α3, and α5 were not involved. Subsequent tests of stiffness-dependency revealed in 2D an increase in the integrins α1, β1, and β3 expressions with decreasing stiffness under normoxia and also an increase in the expression of α1, β1, and β3 integrins with decreasing stiffness under hypoxia. However, the extent of increase was lower in hypoxia. In 3D, the study showed an increase in the expression of the α1, β1, and β3 integrins with decreasing stiffness under normoxia, similar to 2D, but a decrease in the expression of the α1, β1, and β3 integrins with decreasing stiffness under hypoxia [196]. Another study with much higher substrate elastic modulus values in the MPa range [195], in contrast to the above cited study in the kPa range, cultivated hMSCs and hCHs in 2D on hydrogels with elastic modulus values of 0.8 MPa, 4.7 MPa, 223.7 MPa, and 309.9 MPa. This study demonstrated a stiffness- and cell type-dependent expression because in hMSCs the tested expression of the integrin subunits α1, α2, α5, αv, β1, and β3 was stiffness-regulated. In CHs, the integrin subunits α1, α2, αv, β1, and β3 were stiffness-regulated. However, the subunits α1, β1, and β3 displayed a strong response, whereas α5 was not stiffness-regulated. Furthermore, this study silenced (only) the integrin subunit β1 in MSCs because that subunit mediates SOX9 and runt related transcription factor 2 (RUNX2) expression and silencing abolished mRNA expression. Combining the data from two studies [195,196], one can conclude that hMSCs appear to be elastic modulus-sensitive in the range from 2 Pa to 309.9 MPa and respond with differential α1, β1, and β3 expression. Moreover, a stiffness-dependent integrin subunit expression in both hMSCs and hCHs illustrates how material stiffness gives rise to differential FA compositions in these two cell types.

## 16. Differential MSC Behavior in 2D vs. 3D

To assess MSC differentiation in a 3D environment with low stiffness for inducing chondrogenic differentiation without use of exogeneous differentiation supplements, one study altered the composition and the mechanical properties of collagen-glycosaminoglycan scaffolds [197]. Using substrates with elastic moduli of 0.5, 1.0, and 1.5 kPa and different glycosaminoglycan (GAG) types, they demonstrated that scaffolds with a relatively low elastic modulus of 0.5 kPa significantly upregulated SOX9. The chondrogenic differentiation of rMSCs induced by a soft 3D environment as seen in a study by Murphy et al. [197] is in general agreement with findings in the 2D study reported above. Hence, hMSCs cultivated in 3D using hydrogels with a lower elastic modulus (3.5 kPa) caused the cells to undergo chondrogenesis [198], whereas hydrogels with a higher elastic modulus (53.6 kPa) induced hypertrophic marker expression (collagen type X, matrix metallopeptidase 13 (MMP-13)) and osteogenic differentiation marker expression (alkaline phosphatase (ALP)) of hMSCs with increased MMP-13 and type X collagen and ALP. This expression was not modulated by ROCK but by myosin II, as blocking ROCK with Y27632 had no obvious effects, and using blebbistatin for inhibition of myosin II reduced the expression of MMP-13, type X collagen, and ALP in high crosslinking density, stiff constructs [198]. Thus, these data are in accordance to rMSC studies on stiff substrates in 2D, which also demonstrated osteogenic differentiation marker upregulation [152]. However, this comparison is difficult, as the substrates used in both studies had comparable stiffnesses but different biomaterial types. Regardless, we identified one study that assessed hMSC chondrogenesis on the polymers’ gelatin, chondroitin sulfate, hyaluronic acid, and polyethylene glycol in 2D vs. 3D [199]. This study demonstrated that the expression levels of the chondrogenic differentiation markers collagen type II, ACAN, and SOX9 were comparable in 2D vs. 3D but much higher in 3D, and this behavior was observed for all four polymers. Increased chondrogenesis was always accompanied by enhanced N-cadherin expression, suggesting N-cadherin as a robust marker to, for example, select culture conditions that promote chondrogenesis. Interestingly, in another study [199], ROCK inhibition had minimal effects in the 2D or 3D models and varying the polymer used did not change the chondrogenic response to ROCK inhibition within each culture model. However, ROCK inhibition decreased chondrogenesis in a newly developed gelatin-based microribbon (μRB) model, which is a highly macroporous scaffold, in which encapsulated cells attach to the surface of individual μRBs and exhibit rapid cell spreading upon encapsulation [200]. Thus, it would be expectable that ROCK inhibition would decrease chondrogenesis in this model, as highly spreading cells are known to contain relatively high amounts of activated ROCK and more pronounced stress fiber formation [136]. In turn, the limited effects of ROCK inhibition on chondrogenesis in the 2D or 3D models in previous research [199] can perhaps be explained by the limited cell spreading known to occur in hydrogels [201].

## 17. Immuno-Modulative and Angiogenic Role of Material Stiffness in MSCs

In 2011, Caplan and coworkers proposed that MSCs are released during injury from their perivascular location, become activated, and establish a regenerative microenvironment by secreting bioactive molecules and regulating the local immune response [202]. Moreover, they termed these trophic and immunomodulatory activities as site-regulated “drugstores”. Thus, it is thought that the main mechanism for MSCs’ beneficial effects in tissue regeneration might be based on their capability to produce a large variety of bioactive trophic factors that stimulate neighboring parenchymal cells to start repairing damaged tissues [203]. Another interesting suggestion given in 2016 was that the number of MSCs required to exert trophic actions might be less than necessary for tissue replacement [204]. In this context, a potential association of material stiffness with trophic activities has generally not yet received much attention. However, recent evidence suggests that material stiffness modulates the paracrine signaling of a few cell types [205,206,207,208] and even the intracellular reactive oxygen species (ROS) level in human adipose-derived MSCs (ADMSCs) [209]. One study varied either poly(ethylene)glycol diacrylate (PEGDA) hydrogel stiffness but kept the cell adhesive sites constant or varied the concentration of the cell adhesive sites. Under these conditions, matrix stiffness but not the available cell-adhesive sites played a critical role in pro-angiogenic signaling of hMSCs [148]. Another study revealed that material stiffness modulates the expression of interleukin-8 (IL-8) as well as vascular endothelial growth factor (VEGF) of hMSCs [210], a potent angiogenic factor, illustrating the pro-inflammatory and angiogenic cues of (increasing) material stiffness in hMSCs.

## 18. The Role of Material Stiffness in Inducing Re-Differentiation of CHs after Serial Expansion-Induced De-Differentiation

A significant problem in AC tissue engineering and scaffold transplantation such as performed in ACI is that scaffolds have to be seeded with a sufficient number of cells prior to surgical transplantation. For this, CHs are expanded to generate high cell numbers through serial passaging. However, this passaging leads to de-differentiation and above a certain threshold it eventually results in fibroblast-like CHs with a fibrogenic phenotype, which limits the amount of serial passages and, thus, the number of available CHs [211,212,213]. Therefore, previous studies investigated how CH de-differentiation can be reversed to generate a healthy hCH phenotype [214] by using alginate bead culture [214], pellet culture [212], agarose hydrogels with varying RGD adhesion site densities and mechanical properties [215], photo-crosslinkable hydrogels [216], chimeric Activin A/BMP2 ligand AB235 [217], serum or growth factor cocktails [218], low oxygen concentrations [219], and MSC co-culture [220]. For the interested reader, factors that are considered particularly supportive of CH expansion and re-differentiation are summarized elsewhere (see [221]).

One study tested the re-differentiation of monolayer-expanded, de-differentiated pCHs in 3D agarose hydrogels with varying RGD adhesion site densities and mechanical properties (3.7 kPa vs. 53.2 kPa) [215]. Unexpectedly, adhesion site availability inhibited re-differentiation and decreased in an RGD dose-dependent manner sGAG production per cell. Similarly, hydrogels with the highest RGD density remained positive for collagen type I and exhibited lowest collagen type II. Softer gels contained higher pCH numbers and ECM amounts after two weeks of culture but, interestingly, substrate stiffness did not affect re-differentiation. These results were interpreted in a way that adhesion site density, but not stiffness, influences pCH re-differentiation in 3D [215]. As discussed above, such findings are difficult to interpret, as the data can also be explained by an adhesion-independent mechanism, in which cells sense cell volume confinement in 3D culture [165]. Another study [222] used very soft hydrogels (2–20 Pa) to investigate the influence of stiffness in 2D and 3D environments on sheep CH (sCH) phenotype but no de-differentiation via serial passaging was performed. The study demonstrated that the softest collagen hydrogels, used as monolayer or 3D culture system, increased the expression of ACAN, collagen type II, and SOX9. The loss of chondrogenic phenotype on stiffer hydrogels correlated with a diffuse organization of actin stress fibers [222]. Here, the 2D experimental results of sCH differentiation were comparable to the results of the 3D environment, as sCH phenotype, morphology and organization of cytoskeleton were comparable across both systems and, importantly, stiffness-mediated. Interestingly, the elastic moduli of these hydrogels used by Sanz-Ramos et al. [222] were much softer (2–20 Pa), compared to the other discussed studies (3.7 kPa vs. 53.2 kPa) [164,215], which did not find any association between chondrogenic mRNA expression and material stiffness. Another study also investigated the re-differentiation of passage de-differentiated CHs and chose infant and adult hCHs from polydactyly patients for culture on transglutaminase cross-linked hyaluronic acid hydrogels with elastic moduli of approximately 2 kPa, 5 kPa, and 8 kPa [223]. This study demonstrated that collagen type II expression and sGAG deposition normalized to DNA content of infant hCHs were not stiffness-dependent (see Supplementary Dataset). Data on adult hCHs or for higher elastic moduli than the investigated relatively low range of 2–8 kPa were not given. Collectively, material stiffness-modulated CH phenotype regulation appears sensitive to a certain stiffness range but only a few studies are available that used serially passaged CHs for re-differentiation across a range of stiffnesses. Thus, the role of material stiffness in inducing re-differentiation of CHs after serial expansion-induced de-differentiation remains unclear.

## 19. The Role of Material Stiffness-Dependent β-Catenin Signaling in CH De-Differentiation

**Figure 4:**
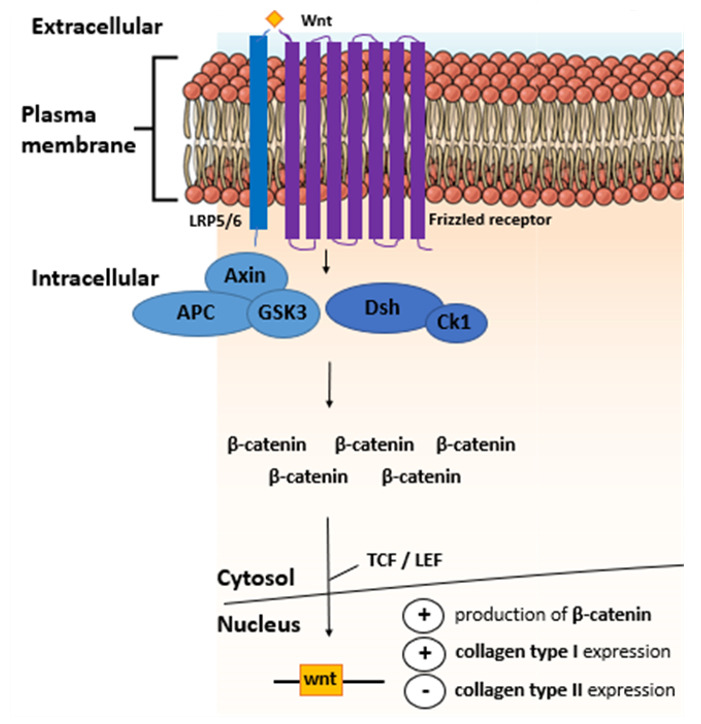
Material stiffness-induced Wnt signaling. Binding of a Wnt ligand to the Frizzled receptor and LRP5/6 receptor activates the Wnt signaling pathway. Wnt causes translocation of Axin and the destruction complex adenomatous polyposis coli (APC) and glycogen synthase kinase 3 (GSK3) to the plasma membrane, inhibiting β-catenin degradation through the destruction complex. Dsh becomes activated and β-catenin accumulates in the cytoplasm. β-catenin translocates into the nucleus and promotes target gene expression of Wnt genes through binding TCF/LEF co-activators and further production of β-catenin. Increasing β-catenin leads to higher expression of type I collagen and decreased expression of type II collagen in CHs.

One of the molecular mechanisms involved in stiffness sensing is the Wnt/β-catenin signaling pathway (Figure 4). The Wnt/β-catenin pathway is responsible for many cell functions such as adhesion, migration, differentiation, and proliferation [224]. A study demonstrated that relatively high material stiffness enhanced the expression level of several members of the Wnt/β-catenin pathway in both MSCs and primary mCHs [225]. In this study the accumulation of β-catenin, an intracellular signal transducer of the Wnt signaling pathway, was increased by the integrin/FAK pathway due to high material stiffness. Accumulated β-catenin binding to the Wnt promoter region acted in a positive feedback loop, which plays a significant role in mediating Wnt signaling on stiff ECMs. Another study reported that the nuclear accumulation of β-catenin and subsequent stimulation of β-catenin-Tcf/Lef transcriptional activity causes de-differentiation of the articular CHs of two-week-old New Zealand white rabbits, characterized by decreased type II collagen expression and initiation of collagen type I expression [226]. Moreover, α-catenin blocks the β-catenin-mediated inhibition of collagen type II expression in these rabCHs [227] through a direct interaction between α-catenin and β-catenin [226] and, thus, increases collagen type II expression. These studies highlight how increased material stiffness contributes to CH de-differentiation through increased β-catenin nuclear accumulation. In this context it is noteworthy to mention that elevated levels of β-catenin have been detected in human OA knee joint cartilage [226].

## 20. Collagen Type II Fragment Production and Subsequent Catabolic Effects are Modulated by Rho/ROCK Activation in CHs

Interestingly, many clinical biomaterials for AC repair consist of collagen type I and/or type III, (e.g., as a fleece [228,229], sponge [230,231,232,233,234], gel [235], membrane [236,237], or matrix [235,238]) that in some cases are substituted with other materials. hCH culture on collagen type I or II promotes matrix production and turnover without significant differences between collagen types I and II, indicating that the use of collagen type I or II coating for in vitro models appears to be a sound basis for in vivo repair procedures [239]. Nevertheless, the predominant usage of collagen type I but not type II for clinical biomaterial production is likely connected to the fact that collagen type II fragments containing the N- and C-terminal telopeptides have dose-dependent catabolic activities similar to fibronectin fragments and increase the production of NO, cytokines, and MMPs in pCHs [240]. Moreover, in both bCHs and hCHs collagen type II fragments inhibit collagen synthesis, which has been shown in hCHs to be dose-dependent [241]. In human AC explant collagen type II fragments perturb AC homeostasis, as the fragments suppress collagen synthesis [242] and upregulate catabolic processes leading to a net loss of tissue mass [241].

In the context of this review one study exposed epiphyseal rCHs to transforming growth factor α (TGF-α), which inhibits articular chondrocyte anabolic capacity, increases catabolic factors, and contributes to the development of chondrocyte clusters [243]. Specifically, TGF-α induced actin cytoskeleton modulation, altered cell morphology, RhoA/ROCK, MAPK/ERK kinase, PI3K, and p38 MAPK signaling and downregulated collagen type II, ACAN, and SOX9 expression [243]. Moreover, collagen type II and ACAN cleavage fragments were induced with TGF-α. Importantly, fragment production was greatly reduced by inhibiting MEK/ERK and Rho/ROCK activation, demonstrating a link between Rho/ROCK activation and collagen type II fragment generation. As Rho/ROCK activity increases with substrate stiffness [107] and ROCK plays a key role in the stiffness sensing ability of CHs [108], one could theoretically ask whether collagen type II fragment generation and subsequent catabolic effects might be material stiffness-sensitive. However, such data is not available to the knowledge of the authors. Another potential way to link fragment generation to material stiffness might be that the epidermal growth factor receptor (EGF) receptor, which binds fragment-inducing TGF-α, is relevant for stiffness sensing and increases spreading and contractility on stiff, but not on soft substrates [244]. Thus, the TGF-α-binding EGF receptor is stiffness-sensitive but whether such mechanisms contribute to material stiffness-dependent catabolically acting collagen type II fragments has not yet been demonstrated.

In MSCs, the collagen type II supports chondrogenic differentiation, whereas collagen type I suppresses collagen type II expression and chondrogenic differentiation [245,246,247,248]. However, no data in a material stiffness-dependent context were found. In summary, the role of material stiffness in the generation of catabolically acting collagen type II and other ECM fragments has not been addressed sufficiently.

## 21. Biomaterials Used for Clinically Inducing Human AC Repair

Clinical scaffolds for cell-based or cell-free therapies should induce or stabilize a chondrogenic phenotype in both CHs and MSCs. Their use in AC repair surgery should not be too complex, facilitate the implantation of the cells, and fill the AC defect. In addition, the scaffolds have to be bio-compatible, non-toxic, resorbable, and withstand the mechanical demands within the joint. A list of the available biomaterials that have been used or are in use for clinical AC repair is given in Table 1 and Table 2. The following text section reviews what is known about CH behavior in regard to these biomaterials but does not review the clinical performance nor assess the clinical value.

Not many studies are available that assessed basic science parameters in the context of this review. One interesting study assessed the influence of scaffold architecture on the CH distribution and behavior [228] by comparing matrix-associated CH transplantation grafts such as Hyalograft^®^ C autograft (Fidia Advanced Biomaterials, Italy), a hyaluronan web, Chondro-Gide^®^ (Geistlich Biomaterials, Switzerland), a collagen type I/III fleece, CaReS^®^ (Arthro Kinetics Biotechnology GmbH; Austria), a collagen type I gel, and Novocart^®^ 3D (TeTeC, Germany), a collagen type I sponge containing chondroitin-sulfate. The study found that the hCHs formed cell layers, nests, or clots in the hyaluronan web, and subconfluent or confluent layers or multilayers in the collagen fleece, whereas the CHs were not situated in any groups in the collagen gel. In the collagen sponge, only a few hCHs built local aggregates and most hCHs were situated as singles, suggesting that cell–cell contacts occur in the hyaluronan web and in the collagen fleece but not in the collagen gel or sponge. In the context of AC repair with hMSCs, it might be noteworthy that a direct cell–cell contact of hMSCs with hCHs is considered a key mechanism in multipotent MSC-mediated chondrogenesis [253]. Thus, should one consider investigating these four materials, which are intended to be used with CHs, for future MSCs or CH-MSC co-culture-based AC repair instead; it would be important to keep in mind that not all materials allow cell–cell contacts equally.

The above discussed study also showed that hCH morphology was mainly elongated and polygonal in the hyaluronan web; largely polygonal in the collagen fleece; spherical, elongated, and polygonal in the collagen gel; and mainly spherical in the collagen sponge. This is interesting, as a spherical shape indicates a differentiated phenotype, whereas an elongated shape indicates a more de-differentiated phenotype [254,255]. Equally interesting is the fact that comparable pCH morphologies have been previously observed (see [166]). This study assessed how matrix elasticity influences pCH phenotype and demonstrated a round cell morphology and chondrogenic expression profiles on relatively soft PAA hydrogels and a spread morphology with decreased collagen type II and increased ACAN and collagen type I expression on harder PAA hydrogels. Thus, one would expect that clinically used biomaterials for AC repair that are associated with distinct CH morphologies would also be associated with distinct differences in the expression profiles of adhering CHs. Indeed, in a subsequent study, Nuernberger et al. [228] then compared the mRNA expression profiles of hCHs on the four materials [256] and found that Novocart^®^ 3D hCHs displayed the lowest collagen type I expression, whereas collagen type II expression levels were comparable between Hyalograft^®^ C autograft, CaReS^®^, and Novocart^®^ 3D. Interestingly, the collagen type II to I ratio was comparable between Hyalograft^®^ C autograft and Novocart^®^ 3D but higher in CaReS^®^. This ratio presents the balance between a functional chondrocyte phenotype, as found in intact AC, and a modulated proliferative in vitro phenotype, and can easily be used for comparing cell phenotypes across biomaterials or across cell sources [214]. Interestingly, these studies demonstrated that the biomaterial that was associated with a mostly spherical hCH morphology, namely Novocart^®^ 3D, was also characterized by the lowest collagen type I expression level, highlighting the primary role of cell shape in the modulation of the CH phenotype [145,257]. The material with a relatively high collagen type II to I ratio was CaReS^®^, which can be explained by the fact that monolayer expansion is not being used in this system [258]. Collectively, these data give rise to the question(s) whether the observed differences in CH morphology and the associated expression profiles might be related to differences in material stiffness, as the previous text sections of this review clearly demonstrate a correlation of material stiffness with resulting phenotype. However, no data on material stiffness or other mechanical parameters for the materials listed in Table 1 have been found by the authors.

## 22. Discussion

The aim of this review was (i) to summarize the current knowledge on how cells perceive and transduce material stiffness, and to answer the question whether the approach to control material stiffness for guiding cell fate is effective in the context of CH phenotype and MSC differentiation, as those cells remain the most relevant cell types for clinical cartilage repair. Moreover, we reviewed the literature (ii) to elucidate if the biomaterials that have been used or are being used for clinical cartilage repair are known to utilize material stiffness for controlling cell functions. An important insight produced by this review is that both CHs and MSCs are highly susceptible to material stiffness, as CH morphology [167,169], proliferation [164], clustering [164], and phenotype [163,166,167], MSC migration [139,140], proliferation [121], morphology, lineage determination and differentiation [44,259] as well as certain immuno-modulative and angiogenic roles of MSCs [148,210] are stiffness-mediated. Thus, controlling material stiffness for guiding cell fate is undoubtedly an effective approach for experimentally controlling CHs and MSCs. Arguably, this approach would also be a promising strategy for biomaterials used in the context of clinical cartilage repair but, surprisingly, this review found that information on the material stiffness of currently or previously used clinical biomaterials was not available. This suggested that clinical cartilage repair biomaterials could not have been designed with the concept in mind to control material stiffness for steering cell fate. However, the limited basic science data that is available on these biomaterials suggest that the elicited effects on CHs through a combination of material properties and architecture are effective in modulating CH phenotype. However, the authors believe that intentionally using the parameter ‘material stiffness’ as a cell-instructive cue is a not yet seized opportunity for developing novel clinical biomaterials for the future of AC repair.

The cell morphology and mRNA expression profiles of CHs adhering to graft residues of clinically used biomaterials at the time of implantation have been sufficiently investigated to allow comparisons to other studies on cell morphology and mRNA expression profiles that used non-clinical biomaterials. The rationale was to use cell shape and mRNA expression profiles as markers for the effects of material stiffness, as both cell morphology and expression are mediated by material stiffness [143] but also by scaffold architecture [260,261] and biomechanical cues [147]. For example, decreasing diameters of electrospun chitosan fibers upregulate the mRNA expression of collagen type II in CHs [262]. In the above discussed residues of grafts used for clinical cartilage repair [228], the CHs encompassed a range of morphologies such as an elongated shape as a marker of a de-differentiated CH, a polygonal shape as a marker of an intermediate phenotype, and a spherical shape as a marker of a fully differentiated phenotype. Interestingly, the biomaterial that was associated with a mostly spherical CH morphology was also characterized by the lowest collagen type I expression level, highlighting the important role of cell shape in modulating CH phenotype [106,174]. Moreover, the material with a relatively high collagen type II to I ratio contained a not further specified mixture of spherical, elongated, and polygonal CH shapes. Associating a specific cell shape with this expression profile was not possible, as the authors of this review cannot pinpoint what type of CH shape contributed most to the reported mRNA expression levels. However, these observed CH morphologies and associated mRNA expression profiles were comparable to those that were present on softer experimental substrates, which induced a chondrogenic CH phenotype [163,166,167,222]. Despite this agreement, it is impossible to relate the reported effects of the investigated clinical biomaterials on CH shape and expression to material stiffness, as we do not know the material stiffnesses of these biomaterials. Furthermore, not only their stiffnesses but also their topographies [260,261] would likely have contributed to modulating CH shape and expression profiles.

**Figure 5:**
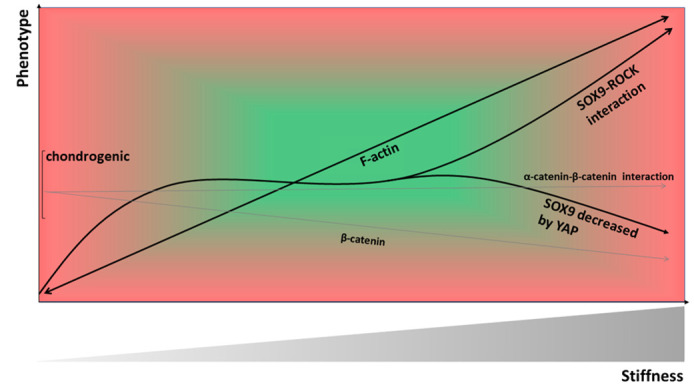
Model of the material stiffness-dependency of CH phenotype. The most prominent signaling pathway involved in stiffness sensing is the RhoA/ROCK pathway. For CHs, a direct ROCK–SOX9 interaction can explain the stiffness-mediated effects on CH phenotype, as SOX9 is a potent chondrogenic transcription factor. With increasing stiffness RhoA/ROCK induces more actin polymerization and stress fiber formation, which has an inverse correlation with CH differentiation in some studies and in others RhoA/ROCK signaling appears to act both pro- and anti-chondrogenically. We suggest resolving this apparent contradiction of the differential effects of RhoA/ROCK on CH phenotype by suggesting that the effects of a direct ROCK–SOX9 interaction define the CH phenotype at sub-chondrogenic and chondrogenic stiffnesses and that the stress fiber-inducing effects of ROCK and subsequent induction of de-differentiation define CH phenotype at supra-chondrogenic stiffnesses. The YAP/TAZ pathway is also regulated by material-stiffness. High material stiffness acts through increased YAP expression and nuclear accumulation as a negative regulator of a healthy chondrogenic CH phenotype. As already mentioned, we suggest a CH phenotype-defining effect of ROCK–SOX9 interaction at sub-chondrogenic and chondrogenic stiffnesses, whereas not only the stress fiber-inducing effects of ROCK, but also the increased YAP expression and nuclear accumulation define the degenerative CH phenotype at supra-chondrogenic stiffnesses. This assumed association between YAP and SOX9 would require increasing levels of YAP expression and nuclear accumulation with decreasing levels of SOX9. Another material-stiffness regulated mechanism is the α-catenin–β-catenin interaction. Increasing material stiffness leads to elevated nuclear β-catenin accumulation and subsequent CH de-differentiation. In addition, α-catenin is upregulated with increasing material-stiffness and can block β-catenin-mediated inhibition of collagen type II expression. The authors suggest a potential connection of α-catenin, β-catenin, YAP, and SOX9 on CH phenotype. Moreover, α-catenin also regulates actomyosin contractility and can recruit actin and vinculin through hidden binding sites. This promotes further actin polymerization and subsequent nuclear YAP localization, which decreases SOX9 expression in CHs. Thus, material stiffness-triggered increases of β-catenin and theoretically also a material stiffness-triggered interplay of α-catenin, YAP, and SOX9 may impact CH phenotype.

It is noteworthy to mention that ACI is recommended for isolated, focal AC defects [31,263]. Thus, future clinical biomaterials would be used in part for the phenotype stabilization of healthy CHs and to control the differentiation and immuno-modulative functions of MSCs in non-degenerative joints. In this context, this review has collected ample evidence to suggest that controlling material stiffness for guiding CH and MSC fate and functions is a highly effective approach. However, recent studies have revealed that ACI is not only being used for isolated, focal AC defects. In 34% to more than 60% of cases, ACI is also used for treating degenerative AC defects, as graded by the treating physician at the time of AC repair [264,265]. This is relevant for future clinical biomaterials that would utilize material stiffness as a cell-instructive stimulus, as those numbers raise the question whether material stiffness could also be used in a degenerative context. However, it appears that the role of material stiffness in an OA-related degenerative context has not been sufficiently investigated, for example, by systematically assessing the effectiveness of stiffness-induced re-differentiation of de-differentiated, serially passaged CHs, or by investigating OA CHs in this context. Another point to consider is that CHs for clinical ACI are usually derived from a standard location such as the knee joint intercondylar notch. Nevertheless, other cell sources such as CHs derived from AC lesions [214], from the knee joint trochlea [266], and from dissected AC fragments in joints with osteochondritis dissecans [267,268], as well as CHs within their native pericellular matrix termed ‘chondrons’ [269] are investigated as additional cell sources for AC repair. Given that CH properties across human joints differ in multiple ways [270,271,272,273] and that studies on the response of CHs from these locations to material stiffness are not available, the role of material stiffness for controlling CHs from multiple locations has not yet been investigated. In contrast, the few studies on the pro-inflammatory and angiogenic cues of (increasing) material stiffness in MSCs are promising because they suggest a relevant modulatory role of material stiffness. Collectively, because CHs and MSCs are highly susceptible to material stiffness, the authors of this review speculate that a more targeted use of the material stiffness for developing novel clinical biomaterials will greatly improve controlling CH and MSC AC regenerative properties for the future of cartilage repair. In this context, and based on the available data across various species and biomaterials, the induction and/or stabilization of a chondrogenic phenotype in CHs appear to be promoted by relatively soft 2D substrates of 4 kPa to ≥10 kPa [122,163,166,222], as those induce a round mCH morphology [163], maintain a CH phenotype indicated by a higher expression of collagen type II, ACAN, SOX9, and lower expression of collagen type I [166,222], and decrease YAP expression and cytoplasmic YAP accumulation [122]. Comparisons with studies that used stiffer substrates are difficult when the stiffness ranges do not overlap (e.g., comparing these studies to [108]). In 3D the stiffness values (e.g., for CHs GAG accumulation) appear much lower. Similarly, soft substrates appear suitable for MSC culture because substrates with 3.5 kPa induce chondrogenesis (in 3D) [198] and with 2 kPa maintain low levels of Il-8 expression. The corresponding “ideal” molecular signaling levels supporting a chondrogenic CH phenotype have been integrated into a model of the material stiffness-dependency of CH phenotype (see Figure 5).

This review introduced the cytoskeletal structures, mechanosensitive proteins, and molecular pathways that are known to be involved in stiffness sensing to the AC-focused reader (Table 3). The involved mechanosensory mechanisms range from individual proteins or protein assemblies to the cytoskeleton and the nucleus. Two mechanosensitive proteins, namely talin and vinculin, play key roles in mechanotransduction [64,65] because conformational changes translate mechanical deformation of for example, talin, into biochemical reactions by revealing otherwise hidden binding sites for additional partners. It has been demonstrated that rCHs express vinculin [180], that both bCHs and mCHs express talin [185], and that hCHs express layilin, a talin-binding receptor, which, interestingly, is downregulated by interleukin-1β [274]. However, in terms of specific mechanosensitive molecules not much else has been investigated in CHs or in MSCs. Multiple signaling pathways are involved in stiffness sensing (Table 3), of which the RhoA/ROCK pathway is perhaps the most prominent (Figure 2), as this pathway is a central regulator of MSC fate and CH phenotype. For example, MSC lineage commitment towards certain directions can be controlled by material stiffness [44,94,151,152,153] but also by growth factors [121] or by generating specific cell shapes, using microcontact-printed adhesion sites in conjunction with induction media [136,188]. Regardless, a common mechanistic denominator in 2D systems appears to be the modulation of endogenous Rho GTPases signaling [121,136,188,275]. In the context of CHs, a direct ROCK–SOX9 interaction can explain some effects of ROCK on CH phenotype because SOX9 is a potent chondrogenic transcription factor [191]. In strong contrast, RhoA/ROCK also induces actin polymerization and subsequent stress fiber formation [104,192,193]. It has also been demonstrated convincingly that RhoA/ROCK exhibits an inverse correlation with CH differentiation [171]. Consequently, one must note (i) that RhoA/ROCK signaling appears to act both pro- and anti-chondrogenically, and (ii) that stiffness sensing appears to play a significant role in this balance between these pro- and anti-chondrogenic effects, as ROCK activity is material stiffness-dependent. Thus, we resolve this apparent contradiction of the differential effects of RhoA/ROCK on CH phenotype by suggesting that the effects of a direct ROCK–SOX9 interaction define CH phenotype at sub-chondrogenic and chondrogenic stiffness and that the stress fiber-inducing effects of ROCK and subsequent induction of de-differentiation define CH phenotype at supra-chondrogenic stiffnesses. Thus, the available molecular signaling data were integrated into a stiffness-regulated CH phenotype model, which is illustrated in Figure 5.

Moreover, material stiffness also impacts proliferation, as pathways involved in stiffness sensing modulate S-phase entry. For example, stiff substrates foster stress fibers, a spread morphology which, in turn, promotes the proliferation of many cell types [121,127] including AC CHs [122] and MSCs [121]. This link between morphology and induction of proliferation is relevant, as it might theoretically allow controlling the proliferation rate that is desired for a specific time frame via a tunable material stiffness (e.g., via optogenetics) [276]. In contrast, biomaterials could also be developed to suppress early proliferation such as seen in early OA [277] if desired. Stiff substrates also promote active YAP/TAZ in the nucleus, a transcription co-activator, which promotes the proliferation of multiple cell types [127] but not of hMSCs [121] or AC rCHs [122]. Thus, another relevant signaling pathway is the YAP/TAZ pathway, as it not only inhibits proliferation in MSCs and CHs but also controls hMSCs lineage commitment [155] mediated in part via substrate mechanics-regulated cell spreading [156], and because it contributes to regulating AC homeostasis through mediating Hippo signaling [162]. In terms of chondrogenesis, YAP is a negative regulator of chondrogenic differentiation of MSCs [157]. Moreover, YAP inactivation is conducive to the maintenance of the chondrogenic phenotype [122] because YAP downregulation on soft substrates helps maintain CH phenotype, and because relatively stiff substrates of 40 kPa increase YAP expression and YAP accumulation in rCH nuclei, concomitant with high expression levels of collagen I and almost no collagen type II expression. Thus, relatively high material stiffness fosters a degenerative CH phenotype through increased nuclear YAP. To explain, according to Dasgupta and McCollum [278], stiffer substrates lead to more robust assembly of FAs and stress fibers, increased activation of the FAK kinase, increased cell spreading, and increased YAP/TAZ activity in a manner that depends on the tension-sensing focal adhesion protein talin, based on previous studies [71,279]. Moreover, another study [280] suggested that YAP/TAZ activation by integrin-dependent FA formation may be linked to the activation of the RhoGEF β-PIX, the small GTPase Rac1, and its effector p21-activated protein kinase (PAK), based on previous research [281,282]. In particular, one study [281] connected β1 integrin-dependent Rac/group I PAK signaling to the activation of YAP1 [281]. In conjunction with other research [195,196], which found that a differential α1, β1, and β3 integrin expression is stiffness-dependent in hMSCs and in hCHs, these studies illustrate how material stiffness might activate YAP signaling in an integrin-Rac-dependent manner. Together, these studies explain how high material stiffness acts through increased YAP expression and nuclear accumulation as a negative regulator of chondrogenic differentiation of MSCs, as described by Karystinou et al. [157], and of a healthy, chondrogenic CH phenotype, as seen elsewhere [122]. Consolidating the material stiffness-dependent effects of both RhoA/ROCK and YAP on CH phenotype, we suggest that the effects of a direct ROCK–SOX9 interaction define CH phenotype at sub-chondrogenic and chondrogenic stiffnesses, whereas not only the stress fiber-inducing effects of ROCK but also the increased YAP expression and nuclear accumulation subsequently define the degenerative CH phenotype at supra-chondrogenic stiffnesses (see Figure 5). In line of this thought, such an assumed association between YAP and SOX9 would require that increasing levels of YAP expression and nuclear accumulation were associated with decreasing levels of SOX9. To the best knowledge of the authors, no systematic study has assessed this association. However, in support of our assumption, a study has reported decreased SOX9 expression levels concomitantly with increased YAP expression and YAP accumulation in the nucleus of rCHs on 40 kPa stiff but not on 4 kPa soft substrates [122]. Another study on growth plate CHs also reported that high levels of phosphorylated YAP accompanied low SOX9 expression levels [283]. In addition, in support of our assumption, another study on esophageal squamous cell carcinoma cell lines found that SOX9 is, at the same time, a downstream target as well as an upstream regulator of YAP signaling as they reported increased YAP protein levels after SOX9 knockdown [284]. However, although it is intriguing to explain a degenerative CH phenotype on stiffer substrates by increased YAP expression and nuclear accumulation and subsequently decreased levels of SOX9, together with stress fiber-inducing effects of ROCK and subsequent de-differentiation, further studies on this topic are needed.

TGF-β1, as illustrated in Figure 3, plays major roles in stiffness-dependent hMSC chondrogenic differentiation [121], CH phenotype regulation [108], and mediating pro-fibrogenic activities during OA progression [285]. Given these roles, and given how aging, mechanical stress, and inflammation contribute to altered TGF-β family signaling [286], a material stiffness-dependent mechanism of the TGF-β receptor is rather interesting. The TGF-β receptor appears to be cell surface tension-sensitive, as altering cellular tension through ROCK inhibition or through cell culture on substrates with varying stiffness leads to a collapse of the physical separation of the receptor complexes TβRI and TβRII within the FAs and leads to multimeric TβRI/TβRII [137]. Under such circumstances, TβRI and TβRII are no longer physically separated and, instead, they interact, which subsequently induces changes in TGF-β-induced downstream effects (e.g., Smad3 activation). Although such effects have not been demonstrated in CHs, it appears appealing to consider the discrete spatial organization of TGF-β receptors in the context of ageing or onset of OA. As discussed previously [277], it is commonly suggested that ageing or onset of OA switches the receptor in TGF-β signaling from the classical activin receptor-like kinase 5 (ALK5)/TGF-β-RI activated Smad2/3 signaling to TGF-β-RI family member ALK1/ACVRL1 induced SMAD1/5/8 signaling, which converts TGF-β function in AC from an anabolic growth factor into a catabolic cytokine [287]. However, to what extent material stiffness-dependent effects might be responsible remains to be seen.

The last signaling pathway that this study discusses is the Wnt/β-catenin pathway. Through the integrin/FAK pathway, material stiffness induces β-catenin nuclear accumulation [225]. This nuclear accumulation of β-catenin and the subsequent stimulation of transcriptional activity cause rabCH de-differentiation [226]. Thus, the Wnt/β-catenin pathway represents another material stiffness-regulated mechanism that impacts on CH phenotype. Interestingly, α-catenin can block the β-catenin-mediated inhibition of collagen type II expression in rabCHs [227] through a direct interaction of α-catenin with β-catenin in the nucleus [226], which experimentally reestablishes CH collagen type II expression. Thus, Figure 5 illustrates the stiffness-mediated effects of β-catenin on CH de-differentiation. Theoretically, recent findings on α-catenin, β-catenin, YAP, and SOX9 might suggest that the discussed effects of these proteins on CH phenotype are connected. Although such data have not been demonstrated in CHs or MSCs, α-catenin regulates the actin-myosin contractility of cardiomyocytes, which controls YAP nuclear accumulation [288]. α-Catenin is upregulated by high material stiffness in Madin–Darby canine kidney (MDCK) cells [289] where it recruits actin and vinculin through a force-dependent cryptic vinculin-binding-site [290]. In turn, vinculin reinforces FAs and nucleates actin polymerization [291], which also promote YAP nuclear localization in cardiomyocytes [292]. As discussed, increased YAP expression and YAP accumulation occur in the nucleus of rCHs on stiff substrates and decrease their SOX9 expression [122]. Thus, material stiffness-triggered β-catenin and theoretically also a material stiffness-triggered interplay of α-catenin, YAP, and SOX9 impact on CH phenotype, resulting in de-differentiation in high stiffness conditions. Although designated studies have not yet been performed in CHs or MSCs, older studies have established a strong link between the cytoskeleton and collagen type I and II expression [254,255,257]. Exploring such potential mechanisms in the context of this review might link the regulation of collagen type II expression through α-catenin, β-catenin, and vinculin directly to the cytoskeletal proteins and forces that are involved in CH stiffness sensing.

On a side note, the here reviewed studies were conducted in 33% of all studies on hCHs, in 16% mCHs, 11% pCHs, 8% bCHs, 8% gCHs, 8% rCHs, 8% rabChs, and in 2% cCHs and also in 2% in sCHs. In 7% of all reviewed studies, hMSCs were used and in 23% rMSCs were used. Despite the obvious usage of cells from multiple species, this review found no conflicting data between species, suggesting that CHs across species might share common stiffness sensing-mechanisms and responses. However, this insight is somewhat limited because very few studies used cells from more than one species and, thus, no head-to-head comparisons are available. Nevertheless, we suggest that future studies should not focus on differences across species but rather on differences between healthy vs. de-differentiated CHs and stiffness ranges across magnitudes of differences.

In summary, the here reviewed knowledge on the substrate stiffness-dependent behavior of CHs and MSCs has important implications for utilizing material stiffness as a phenotype-controlling parameter with the aim to create in situ environments for inducing or maintaining a healthy chondrogenic phenotype. Surgical approaches that might benefit include CH-focused methods such as ACI and other CH-focused procedures [30], whose clinical applications are subject to algorithm-based recommendations [31]. Procedures that might also benefit are bone marrow stimulation methods such as microfracture, nanofracture, and AMIC™.

Current clinical biomaterials were devised in a decade, in which biophysical cues such as material stiffness had not yet emerged as essential determinants of cell fate. This is in contrast to the current view, which clearly recognizes the relevance of biophysical factors because those can be equally important as biochemical and genetic factors [293]. Acknowledging this, studies even use terms such as the “rise of mechano-transduction” [294], “mechano-transduction: use the force” [295], and “mechano-transduction: may the force be with you” [296]. Importantly, the available mRNA expression data were derived from the residuals of clinical biomaterials with CHs that displayed mostly no IL-1β expression, which, in turn, suggests that the investigated CHs were derived from non-degenerative joints [256]. Thus, despite not being optimized for using material stiffness as a cell-instructive parameter, we conclude that current clinical biomaterials control CH phenotype well in non-degenerative settings but not to equal extents. In conjunction, this review collected sufficient evidence to recommend using material stiffness for controlling cell phenotype and as a promising design cornerstone for novel future-oriented, cell-instructive biomaterials for clinical high-quality articular AC repair tissue. Since the future of clinical AC repair lies in developing solutions for degenerative AC lesions or joints, the perhaps most important insight is that material stiffness has immuno-modulative and angiogenic roles in MSCs and modulates growth factor- and pro-inflammatory cytokine-induced changes in CHs. Thus, in the future material stiffness may be used clinically to intentionally modulate a degenerative, chronic inflammatory environment, which might lead to phenotype-instructive, inflammatory response-modulating biomaterials for the future of cartilage repair.

## Figures and Tables

**Table 1:** Cell-based biomaterials used for clinical articular cartilage repairs.

Product Name	Type of Material	Stiffness Data	Morphology	Gene Expression	Porosity	Reference
**Chondro-Gide^®^**	3D Hyaluronan web	NA	Some spherical; mainly elongated; polygonal	De-differentiated phenotype	High (up to 200 µm)	[228,229]
**Hyalograft C**	Autologous chondrocytes grown on a 3D hyaluronan-based scaffold	NA	Spherical, elongated, polygonal	Lower ACAN and collagen type II expression	High (up to 200 µm)	[249,250]
**MACI^®^**	Membrane of type I/III collagen	NA	Elongated-fibroblast like cell shape	High collagen type I		[236,237]
**Novocart^®^3D**	Type I collagen sponges with bilayer structure	NA	Mainly spherical	High expression of collagen type II, little collagen type I, X	High (10-100 µm)	[230,231,232]
**NeoCart^®^**	Collagen type I loaded into sponges of same material	NA	NA	NA	NA	[233,234]
**Novocart Inject**	Autologous CHs, hydrogel is a combination of human albumin and hyaluronic acid	NA	NA	NA	NA	[251]
**RevaFlex™ (formerly DeNovo ET^®^)**	Hyaline neocartilage discs composed of allogenic juvenile CHs	NA	NA	NA	NA	[252]

**Table 2:** Cell-free biomaterials used for clinical articular cartilage repair.

Product Name	Type of Material	Stiffness Data	Morphology	Gene Expression	Porosity	Reference
**CaReS^®^**	Collagen type I	NA	Spheroid, many elongated, polygonal	High collagen type II and ACAN	Low	[235]
**MaioRegen^®^**	Collagen type Iand hydroxyapatite	NA	NA	NA	NA	[238]

**Table 3:** Key molecules that regulate the material stiffness-dependency of cell phenotype.

Key Molecule	Cell Type	Phenotype
**ROCK-SOX9**	CH	stress fiber-inducing effect of ROCK leads to de-differentiation of CH phenotype at supra-chondrogenic stiffnesses [190]
**RhoA/ROCK/myosin II**	CH	high material stiffness increases expression of stress fibers, which leads to a downregulation of collagen type II, but upregulation of SOX9 low material stiffness/disruption of actin network restores the chondrogenic phenotype [163]
	MSC	high material stiffness causes high cross-linking density of fibers → stiffness-specific upregulation of distinct lineage genes [121]
	ATDC5	high material stiffness leads to upregulation of SOX9 [190]
**YAP/TAZ**	CH	high stiffness leads to nuclear accumulation of YAP/TAZ and a degenerative CH phenotype [162] YAP inactivation restores collagen type II levels [122]
	MSC	soft substrate leads to YAP/TAZ accumulation in the cytoplasm → no proliferation [127]/chondrogenic differentiation [122] stiff substrate leads to active YAP/TAZ in the nucleus → induces proliferation [127] and osteogenic differentiation [121]
**TGF-β**	CH	low stiffness + TGF-β lead to elevated levels of chondrogenic gene expression [190] higher stiffness + TGF-β increase cell stiffness and lead to higher SOX9 expression [190]
	MSC	differential effects of TGF-β modulated by stiffness soft material stiffness + TGF-β → chondrogenic differentiation [121] medium material stiffness + TGF-β → myogenic differentiation [121]
**Lamin A**	MSC	soft material stiffness induces low lamin-A expression → adipogenic differentiation [173] high material stiffness induces high lamin-A expression → osteogenic differentiation [173]
**Wnt/β-catenin**	CH/MSC	high material stiffness leads to accumulation of β-catenin and de-differentiation of CHs [225]
**α-catenin**	CH	counteracts the β-catenin mediated inhibition of collagen type II expression [227]
**IL-1β** **Rac1/cyclin D1**	CH	elevated levels of IL-1β increase cellular stiffness [184]
	CH/ MSC	high material stiffness leads to upregulation of cyclin D1 mediated by Rac1, inducing S-phase entry and proliferation [125]

## Abbreviations

AC	articular cartilage
ACAN	aggrecan
ACI	autologous chondrocyte implantation
ALP	alkaline phosphatase
AMIC^TM^	autologous matrix-induced chondrogenesis
ATF2	activation transcription factor 2
Arp2/3	actin-related protein 2/3
BMP	bone morphogenetic protein
CHs	chondrocytes
cCHs	chicken chondrocytes
COL1A2	collagen type I alpha II chain
COL2A1	collagen type II alpha I chain
Col10A1	collagen type 10 alpha I chain
CREB	cAMP response element-binding protein
ECM	extracellular matrix
EGF	epidermal growth factor
ERKs	extracellular signal-regulated kinases
FAK	focal adhesion kinase
FAs	focal adhesions
GAGs	glycosaminoglycans
gCHs	goat chondrocytes
GEF	guanine-exchange factor
hASCs	human adipose-derived stem cells
hMSCs	human mesenchymal stem cells
IL-1β	Interleukin 1 β
IRAK	Interleukin-1 receptor associated kinase
JNK	Jun NH2-terminal kinase
LINC	Linker of nucleoskeleton to cytoskeleton
LOX	lysil oxidase
LPL	Lipoprotein lipase
µRB	microribbon
MMP-13	matrix metalloproteinase 13
MSCs	mesenchymal stem cells
mCHs	murine chondrocytes
MLCK	myosin light chain kinase
myosin II	myosin phosphatase II
NSCs	neural stem cells
OA	osteoarthritis
PAA	polyacrylamide
pCHs	porcine chondrocytes
PEGDA	poly(ethylene)glycol diacrylate
rabCHs	rabbit chondrocytes
rCHs	rat chondrocytes
rMSCs	rat mesenchymal stem cells
rNSCs	rat neural stem cells
ROCK	Rho associated protein kinase
ROS	Reactive oxygen species
RUNX2	runt related transcription factor 2
sCHs	sheep chondrocytes
SMAD	SMA- and MAD-related protein
SMCs	smooth muscle cells
sMSCs	synovium-derived mesenchymal stem cells
SOX9	SRY-related HMG box-containing
TAZ	transcriptional co-activator with PDZ-binding motif
TGF-α	transforming growth factor α
TGF-β	transforming growth factor β
TGFR	transforming growth factor receptor
VEGF	vascular endothelial growth factor
YAP	Yes-associated protein
